# Stochasticity constrained by deterministic effects of diet and age drive rumen microbiome assembly dynamics

**DOI:** 10.1038/s41467-020-15652-8

**Published:** 2020-04-20

**Authors:** Ori Furman, Liat Shenhav, Goor Sasson, Fotini Kokou, Hen Honig, Shamay Jacoby, Tomer Hertz, Otto X. Cordero, Eran Halperin, Itzhak Mizrahi

**Affiliations:** 1 Department of Life Sciences, Ben-Gurion University of the Negev and the National Institute for Biotechnology in the Negev, Marcus Family Campus, Beer-Sheva, Israel; 2 Department of Computer Science, University of California Los Angeles, Los Angeles, CA USA; 3 Institute of Animal Sciences, Agricultural Research Organization, Rishon Letziyon, Israel; 4 The Shraga Segal Department of Microbiology, Immunology and Genetics, Ben-Gurion University of the Negev and the National Institute for Biotechnology in the Negev, Marcus Family Campus, Beer-Sheva, Israel; 5 Department of Civil and Environmental Engineering, Massachusetts Institute of Technology, Cambridge, MA 02139 USA

**Keywords:** Ecology, Microbial ecology, Microbiome

## Abstract

How complex communities assemble through the animal’s life, and how predictable the process is remains unexplored. Here, we investigate the forces that drive the assembly of rumen microbiomes throughout a cow’s life, with emphasis on the balance between stochastic and deterministic processes. We analyse the development of the rumen microbiome from birth to adulthood using 16S-rRNA amplicon sequencing data and find that the animals shared a group of core successional species that invaded early on and persisted until adulthood. Along with deterministic factors, such as age and diet, early arriving species exerted strong priority effects, whereby dynamics of late successional taxa were strongly dependent on microbiome composition at early life stages. Priority effects also manifest as dramatic changes in microbiome development dynamics between animals delivered by C-section vs. natural birth, with the former undergoing much more rapid species invasion and accelerated microbiome development. Overall, our findings show that together with strong deterministic constrains imposed by diet and age, stochastic colonization in early life has long-lasting impacts on the development of animal microbiomes.

## Abstract (web summary)

How complex microbial communities assemble through the animal’s life, and how predictable the assembly process is, remains poorly understood. Here, the authors profile the cow gut microbiome from birth to adulthood in animals born in C-section or natural birth and show that chance events early in life have a strong impact on microbiome development.

## Introduction

The rumen microbial ecosystem and its relationship with the ruminant host is a prime example of obligatory host–microbiome relationships^1^. The host is completely dependent on the microbial community that resides in the upper digestive tract to degrade and ferment the ingested plant biomass, which supports more than two-thirds of its energetic requirements^2–7^. Moreover the rumen microbiome composition of the adult cow was found to be connected with many of its host attributes and performance^8–11^. Recent studies, using metagenomic approaches, show that the rumen microbiome genes are associated with feed conversion processes, growth rate, and appetite^12–14^. Despite decades of research of the rumen microbiome, fundamental questions regarding its composition are still standing. Chief among these is how the complex communities assemble through the animal’s life, and how predictable the assembly process is. In this regard, understanding the balance between stochastic and deterministic forces is of great significance^6^. This need stems from the realization that the balance between these forces essentially shapes microbiome structure and function and its understanding will enable us to potentially predict and identify how and when can we interfere in the assembly process of the microbiome in order to modulate its structure and function.

Stochastic historical processes and events, also termed historical contingency, are thought to play a part in the succession and assembly of microbial communities, where early-arriving species can have a negative (hindering) or positive (facilitating) impact on the late-arriving species^15–17^. A relevant example can be found in the rumen of newly born animals, where aerobes and facultative anaerobes consume the existing oxygen leading to the prosperity of anaerobic microbes and decline of aerobic ones^18^.

If historical contingency conditions the development of the microbiome, perturbations at initial stages of community assembly could have effects on its composition later in life. One of the most common early perturbations is the mode of delivery at birth, which changes the initial state from which the microbiome develops^19^. Studies in humans show contradictory results regarding the impact of delivery type on microbiome assembly^20–24^. While Chu et al. and Stewart et al. did not find significant differences caused by C-section, Bokulich et al. and Yasoor et al. have claimed that C-section does, in fact, have an effect on microbial assembly. In addition, Shao et al.^25^ reported that C-section babies are more prone to invasion by opportunistic bacteria, yet this effect dissipated with age but remained significant at the age of 8 months. In mice, the mode of delivery was shown to have only short-term effects^26^. Similarly, studies in ruminants focused only on short-term effects of dietary intervention (up to 20 weeks) on microbiome composition^27–29^ whereas the impact of mode of delivery was not examined. Hence, despite its potential importance, the effect of historical contingency on assembly dynamics and long-term microbiome composition in gut ecosystems is still unclear.

Here we aimed to elucidate the contribution of historical contingency to the assembly dynamics of the rumen microbiome, taking advantage of the high degree of control in husbandry regimes, diets, and rearing for cows. This tight control of growth conditions enabled us to disentangle historical effects from other potential effects on community assembly. Our study was performed using a single early-life intervention with an ecological context, consisting of two alternative modes of delivery, followed by a long-term high-resolution sampling effort performed in a controlled environment. One-third of the animal cohort was delivered via C-section and the rest through the birth canal, creating conditions that exposed the animals to different species pools via the maternal microbiome^19,30,31^. Following this intervention, we documented the microbiome dynamics of 45 animals over the course of up to 3 years, reared under the same conditions and sampled at high resolution, whereby a third of the cohort was sampled over a 3-year period. Using this unique experimental setup, we explored whether this early-life intervention has long-term consequences on the rumen microbiome-assembly process, thus examining the role of historical contingency on microbiome assembly while disentangling it from the deterministic role of age and diet.

## Results

### Experimental design and data collection

**Fig. 1:**
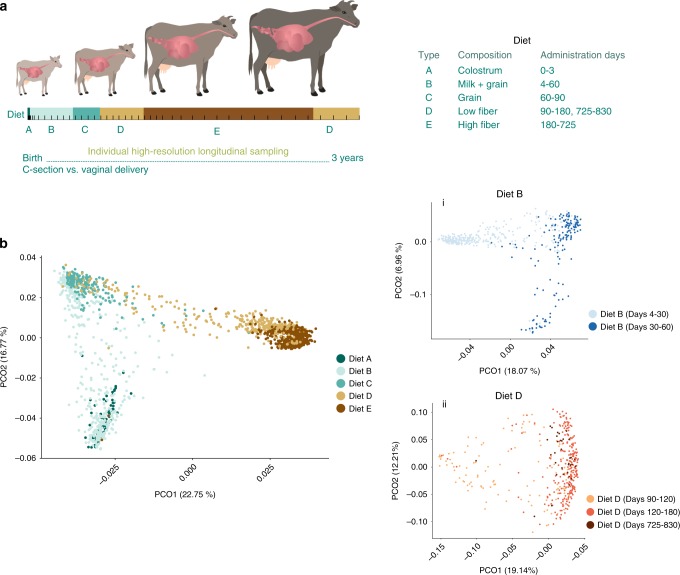
Age plays an important role in community assembly dynamics. **a** The experimental setup consisted of 45 cows (27 delivered vaginally and 18 via C-section), sampled to a minimum of 180 days and over a period of up to 830 days for a third of the cohort (3 years), resulting in high-resolution sampling of more than 1600 samples (bars represents sampling points). The animals were fed with standard dairy-feeding protocols (table on the right), kept under the same conditions, and housed together from the third month of life. See Supplementary Fig. 2 for further details on the sampling regime. **b** Principal coordinate analysis (PCoA) based on Bray–Curtis metrics showed clustering of operational taxonomic units (OTUs) according to age and diet (PERMANOVA, *P* = 0.001, two-sided test). During microbiome development, age-dependent clustering was identified within the same dietary period, where animals were fed with (i) Diet B and (ii) Diet D (PCoA based on Bray–Curtis metrics, PERMANOVA, *P* < 0.05). Source data is provided as a Source Data file.

In this study, our main goal was to reveal the forces that act during the processes of rumen microbiome succession. To that end, we introduced a specific and substantial ecological perturbation at the beginning of life, consisting of two modes of delivery: C-section and vaginal delivery. We compared the rumen microbiome assembly of these cohorts, which had uniform dietary regimes, rearing conditions and very similar genetic backgrounds (all animals were Holstein–Friesian breed and genotyped, Supplementary Fig. 1, see Methods: Animal genotyping). This was based on the assumption that the mode of delivery induces two distinct microbiome-composition starting points that would allow us to study characteristics of the microbial community dynamics throughout life in light of this early perturbation^23,24,30^. We designed a time-series experimental setup with a high sampling resolution of over 1600 samples, consisting of 45 animals, 27 born via vaginal delivery, and 18 via C-section (Fig. 1a, Supplementary Fig. 2). We followed the development of their ruminal microbial community for up to 830 days (all animals except one were sampled for at least 8 months of age, and a third of the cohort was sampled over a 3-year period, with an average and standard deviation of 36 ± 18 samples per animal, respectively). The animals were housed together from the third month of life and kept under similar conditions throughout life (Fig. 1a, Supplementary Fig. 2). During each dietary period, the animals were fed with standard dairy feeding protocols according to their age. As the animals were fed consistently with the same diets over long periods, our sampling regime consisted of multiple sampling during each dietary period, thereby enabling us to distinguish diet and age effects. Our questions focused on microbial species establishment and persistence in the rumen ecosystem, as well as on the forces that govern the microbial succession process.

### Rumen community assembly dynamics is associated with diet and animal’s age

Whereas rumen microbial composition has been previously associated with both diet and age^18,32–34^, the relative contribution of each of these factors is still elusive. Our high-resolution sampling over time enabled us to distinguish between diet- and age-dependent effects. Our analysis showed that both diet and age exert a pronounced effect on microbiome composition (Fig. 1b, Supplementary Fig. 3a; PCoA based on Bray–Curtis metrics; PERMANOVA, *F*_age_ = 20, *F*_diet_ = 6.7, *F*_diet_ × (*F*_age_) interaction =  3.7, *P* < 0.001), with no differentiation between females and males or any effect of gestation on microbiome assembly (Supplementary Fig. 3b, linear regression model). To disentangle the diet × age interaction effect, we focused on temporal changes within specific dietary periods. As animals were kept in homogeneous groups with respect to age and diet, we were able to capture the direct age-dependent effects. This was mainly apparent during two stages of life, in which a constant diet was administered to the animals: during the first 2 months, when the animals were consuming milk and a starter mixture (Diet B), and during the adult stage, when the animals were nearly 3 years of age and consumed a low-fiber diet (Diet D, Fig. 1b, i and ii). Within these dietary periods, a clear and significant clustering according to age was detected (PERMANOVA, *P* < 0.001, in both Diets B and D). Moreover, we could accurately predict the sampling time (age, as month of sampling) based on microbiome composition within each of these diets by applying a random forest classifier (accuracy levels: 0.91 for Diet B and 0.86 for Diet D; Supplementary Data1; see Methods: Similarity measurement). Repetitive and consistent dynamic microbiome patterns were detected by the classifier, which were independent of diet. These diet-independent patterns were also consistent with the changes in the community alpha- and beta-diversity indices, observed during these time periods Supplementary Figs. 4 and 5, arrows).

**Fig. 2:**
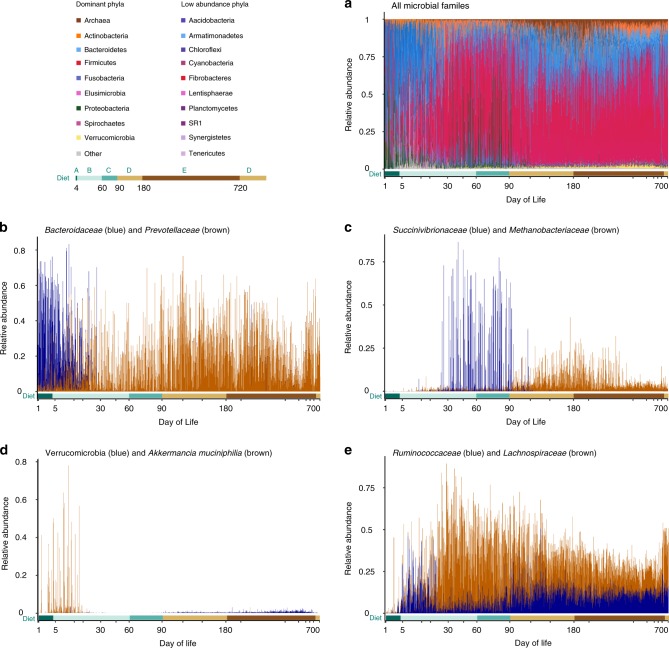
Dynamics of the different microbial families is shaped by age and diet. **a** Relative abundance of 291 microbial families. All families belonging to the same phylum are colored by different shades of the same color. The main phyla are described in the top left corner of the figure. The *Y*-axis represents relative abundance and the *X*-axis represents all of the different samples (*n* = 1634) sorted by sampling day. See Supplementary Fig. 22 for the full family-level analysis. **b** Relative abundance of *Bacteroidaceae* (blue) and *Prevotellaceae* (brown). Both belong to the Bacteroidetes phylum. **c** Relative abundance of *Succinivibrionaceae* (blue) from the Proteobacteria phylum and *Methanobacteriaceae* (brown) from the Archaea domain over time. **d** Relative abundance of *Verrucomicrobiaceae* over time, *Akkermancia muciniphilia (A. muciniphilia*; brown), and all other Verrucomicrobia species (blue). **e** Relative abundance of *Ruminococcaceae* (blue) and *Lachnospiraceae* (brown). Source data is provided as a Source Data file.

When examining the microbial composition between the different diets (Fig. 2a), the Bacteroidetes phylum was more dominant during the first month of life. The *Bacteroidaceae* was the dominant family of this phylum during the first days of life, where the calves were fed colostrum and not supplied with solid feed. Upon introduction of fiber-based diet, the *Prevotellaceae* family increased in relative abundance and became the most dominant family of the Bacteroidetes phylum (Fig. 2b).

As we progress with age and diet, from the starter mixture diet to a low-fiber diet at ~3 months of age, we see a decline of the Proteobacteria phylum (mainly *Succinivibrionaceae)*, followed by an increase of methanogenic archaeal families (*Methanobacteriaceae*), possibly due to the increase in fiber content (Fig. 2c). We speculate that these two events may be interconnected, as several studies have described a negative relationship between these two microbial families, further suggesting that these two groups are co-excluding^11,32,35,36^.

Another aspect characterizing the transition from early to adult stages is the decline of the *Verrucomicrobiaceae* family from 40% to 2–3% at the age of 1 month of the animal and its persistence at this low level of relative abundance until the end of the sampling period. Interestingly a large portion of this family’s abundance is attributed to the species *Akkermansia muciniphila* (Fig. 2d). To the best of our knowledge, there is little or no evidence that this species is present the adult cow rumen while the genus *Akkermansia* has been reported to exist in the gastrointestinal tract of goats and camels^37,38^. *A. muciniphila* is a mucin degrader and an abundant member of the human gut microbiome^39–42^.

The transition from the first to the second month of life was followed by an increase in members of the Firmicutes phylum, which was mainly attributed to the *Lachnospiraceae* family (Fig. 2e), the relative abundance of which was greatly elevated. This microbial family consists of fiber degraders^43^, yet it is unclear why its relative abundance was elevated without any dietary change. This finding would suggest that other environmental factors, driven by microbial niche modification or host control, are involved. Another family of fiber degraders is *Ruminococcaceae*^44^. Although it has been reported that fiber degraders originating from this family are less affected by diet^32^, we clearly see that the transition to higher fiber diet is accompanied by elevated levels of this family (Fig. 2e). In agreement with previous studies^5,18,45,46^, members of these fiber-degrading families appear on the first day of life and increase in relative abundance before the consumption of plant fiber diet. This could suggest a potential inoculation and maintenance mechanism of the rumen ecosystem with these seminal microbial families.

### A set of core successional microbes drives temporal microbiome dynamics

The recurring and consistent pattern of diet- and age-dependent clustering during microbiome development across our sampling cohort suggested that the development of the rumen microbiome is governed by microbes that can consistently be found across animals, diets, and different age periods. Pursuing this hypothesis, we looked for recurring species-level microbial operational taxonomic units (OTUs) across our sample cohort (core successional microbes) and examined whether these exhibit diet- or age-dependent patterns. We identified 2544 core successional microbes, each of them present in at least 80% of the animals, in total representing most of the relative microbial abundance per animal (88% of total relative abundance, Supplementary Data 2). When we examined the contribution of these core successional microbes to microbiome compositional patterns, they better explained the variance and clustering patterns for age and diet than the non-core microbes. This was established by summing the *R*^2^ values for age, diet and age × diet (*R*^2^_core_ = 0.37) derived from a PERMANOVA on the core successional microbes and compared to 1000 iterations of random sets of non-core microbes (average *R*^2^_non-core_ = 0.08).

**Fig. 3:**
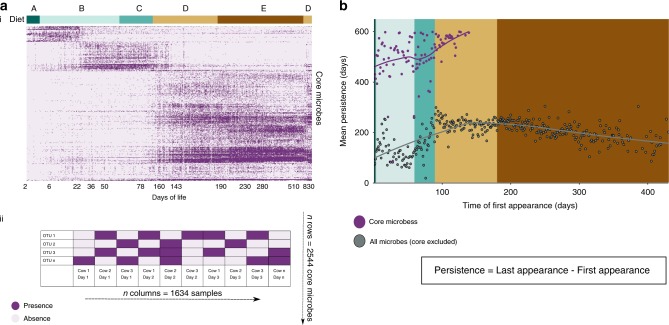
The core successional microbiome persists throughout rumen microbiome development, showing age-dependent shifts. **a** (i) Heat map showing core successional microbes (*n* = 2544) subjected to hierarchical clustering. Diets are indicated in the color-coded bar above. (ii) Graphical illustration explaining the heat map structure. Each row represents a core OTU and each column represents an animal sampled at a specific time. **b** Early-appearing and persistent core successional microbes. Core successional species persistence (*Y*-axis) as a function of time of appearance (*X*-axis). Each dot represents the average persistence (number of days) of all microbes that arrived in the ecosystem on the specified day. Species appearance was measured over a 600-day window from first appearance, and persistence was calculated as the mean of the Δ(*t*_first appearance_ − *t*_last appearance_). Purple dots represent core successional microbes, gray dots represent non-core Microbes. Source data is provided as a Source Data file.

Looking at the distribution of the core successional microbes over time (Fig. 3Ai), we found that they tend to cluster in an age-dependent manner. More specifically, we identified three separate clusters representing three age periods (Fig. 3Ai): (a) the first month of life (days 1–30), (b) the second and third months of life (days 30–100), and (c) the fourth month to third year of life (days 100–830). Moreover, different dietary regimes were fed within these time clusters (Fig. 3Ai). Thus, when we tested for an association of the core successional Microbes with specific diets, we found that 72% of these core successional microbes appear in most diets (Supplementary Fig. 6; see Methods: core Microbes appearance in different dietary regimes). This result indicated that most of the core successional microbes are not diet-specific, and are associated with animal development (Supplementary Fig. 6).

### Core successional microbiome is acquired at early stages and persists throughout life

The dynamic nature of the rumen microbiome composition over time raises questions regarding the forces allowing the core successional microbes to occupy multiple individuals and to persist in this ecosystem. We first sought to characterize each microbial species arrival/invasion time and its persistence during rumen microbiome succession (Fig. 3b). Here, we identified a significant characteristic of the core successional microbiome: these core successional microbes made their first appearance earlier than all others (Fig. 3b, *P* < 0.05, permutation test, see Methods: persistence analysis). Furthermore, core successional microbes were found to arrive only within the first 140 days of life, with most of them introduced on the very first days after birth (days 0–10, see Supplementary Fig. 7) in contrast to the invasion of other microbiome species that occurred throughout the 3 years of our sampling period (see Supplementary Fig. 8).

We next asked whether the time of arrival of a species is related to its persistence within the microbiome throughout life (Fig. 3b). We measured the average persistence of microbes in relation to the day of their first appearance in the rumen ecosystem (Methods: persistence analysis). Interestingly the core successional microbes were three times more persistent than the overall microbiome members, 600 days vs. 200 days, respectively (Fig. 3b, permutation test, *P* < 0.05). Our analysis also showed that microbial persistence has a biphasic dynamics, being overall lower during the early life stages and increasing with age. At 140 days of life, a peak in microbiome persistence was observed, which decreased after this time point and finally stabilized at 200 days of life (Fig. 3b). These results were highly robust as they remained essentially the same even when we increased substantially the stringency of our analysis (Supplementary Fig. 9, see Methods: persistence analysis). Such an outcome suggests that strong ecological selection acts on the rumen microbiome in early life and decreases with time. Notably, these changes occurred independently of the consumed diet. This suggests that arrival and persistence of microbial species is not diet-dependent and could be related to microbial intreactions and age effects. Overall, these findings suggest that microbial persistence is time-dependent and that the core successional microbes that represent most of the microbiome relative abundance are early colonizers that are highly persistent within the rumen ecosystem, potentially due to better adaptation compared to the rest of the microbiome (Supplementary Fig. 10). Family-level analysis showed that the microbial profile of these core successional microbes similar to the overall microbial family profile. This is due to the fact that core successional microbes have a very high relative abundance in the rumen microbiome (88% for all core taxa). When we examined the 10 most abundant core successional microbes, we observed that half of them belonged to the Firmicutes phylum, all of which belonged to the Clostridiales order (Supplementary Data 2). Within this order, we detected species belonging to the *Shuttleworthia* genus and the *Ruminococcaceae* family. The other three species from the Firmicutes phylum were not annotated beyond the order level. Of the other five most abundant species, three belonged to the Bacteroidales order, two of them were classified as *Bacteroides* (genus) and one was *Prevotella* (genus). Another abundant core successional species belonged to the *Succinivibrionaceae* family, which was found to be highly dominant before the transition into high-fiber diets. Surprisingly, the *A*. *muciniphila* species was also found to be one of the 10 abundant core successional species, further highlighting this species as an important member of the rumen successional process.

### Mode of delivery in birth drives historical contingency effects

To this point, our findings showed that the rumen microbiome is shaped by age and diet, both of which are of a deterministic nature. Our experimental design allowed us to also assess the contribution of other types of forces, specifically those that do not necessarily act in a uniform manner across the entire animal population. This was achieved by testing the temporal dynamics of rumen microbiome assembly in light of two distinct modes of delivery which presented a significant early-life interference. First, we asked if indeed the two modes of delivery induce different microbiome starting points. We compared the identities of the microbial species in C-section-delivered and vaginally born animals. We found colonizers that were unique to each of the delivery-mode groups: 1163 microbes in C-section-delivered calves and 2239 in vaginally delivered ones (Supplementary Fig. 11, *P* < 0.05, *χ*^2^ test, see Methods). We then applied different approaches to identify historical contingency-related processes within the rumen microbiome later in life. We identified significant differences in the composition and ecological characteristics of the overall microbiome structure occurring throughout life using different ecological indices, such as species catalog, richness, species evenness, and beta diversity. Vaginally delivered cows showed higher values for these indices, suggesting that the early colonization process affects microbiome development, where the vaginally delivered group has more homogeneous (beta diversity) and even microbiomes (Supplementary Figs. 12, 13, and 14).

**Fig. 4:**
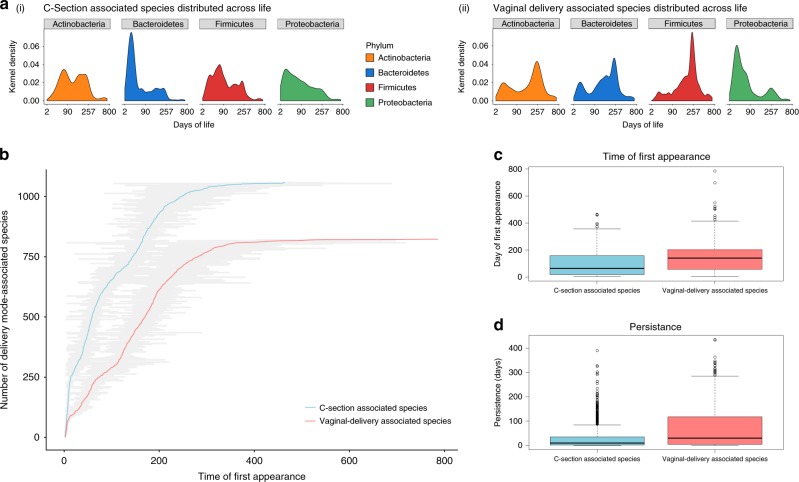
Mode of delivery drives historical contingency effects on rumen microbiome development. **a** (i) Major phylum distribution differs between delivery-mode groups and shows long-term repercussions for mode of delivery. Major phylum kernel density (see Material and methods section for further elaboration of kernel density), depicting significant differences in species (OTUs) count over time, originating from the two delivery-mode groups: C-section (left (i)) and vaginal delivery (right (ii)) (*P* < 0.001; see Supplementary Figures and Results). **b** Early invasion of C-section-associated microbes. Mean ± SE for time of first arrival of delivery mode-associated species. Red line represents average day of appearance of vaginal delivery-associated species (*n* = 809), blue line of C-section-associated species (*n* = 1041), gray bars are SE. *X*-axis depicts the first day of appearance, *Y*-axis depicts the number of delivery mode-associated species arriving in each group. Microbes were ordered by time of appearance. **c** Mean first appearance day for each delivery mode-associated microbe cohort is significantly lower in the C-section cows (*t*-test, *P* = 2.2 × 10–15, two-sided test). **d** Mean maximum time of colonization, depicted as the delta between time of first appearance in any cow to time of last appearance in any cow, is higher in vaginally delivered cows. Colonization time for C-section-associated species is significantly lower (*t*-test, *P* = 2.2 × 10–15, two-sided test). In the data shown as box plots (**c**, **d**),boxes represent the interquartile range (IQR) between the first and third quartiles (25th and 75th percentiles, respectively) and the horizontal line inside the box defines the median. Whiskers represent the lowest and highest values within 1.5 times the IQR from the first and third quartiles, respectively. Source data is provided as a Source Data file.

To further understand these findings, we analyzed our overall dataset, looking for microbes that are significantly associated with delivery mode, and determined how these taxa are distributed across time. We used the Indicator Value (IndVal) method^47^, that measures the association of a certain microbe (a given OTU) to a specific environment. We identified 1850 species-level microbes that were significantly associated with one of the two modes of delivery across time: 809 to vaginal delivery and 1041 to C-section delivery (Supplementary Data 3, Supplementary Fig. 15, see Methods: IndVal). The taxonomic distribution of these delivery mode-associated species was considerably different (Supplementary Fig. 16a). We found in vaginally delivered animals a higher representation of species of the phylum Proteobacteria and lower representation of methanogenic Archaea early in life, as opposed to the Actinobacteria phylum that was underrepresented throughout life. Interestingly, in most cases, the delivery mode-associated species were higher in relative abundance in their group-associated delivery mode (Supplementary Fig. 16b) but exhibited similar dynamics across time in both delivery modes. This general phenomenon can be seen nicely in representative cases of species of the *Peptostreptococcus* and *Dorea* genera that were associated with the C-section delivery mode or species of the *Prevotella* and *Butyrivibrio* genera that were associated with the vaginal delivery mode (Supplementary Fig. 16c). These findings suggest that the dynamics for a given species are governed by global forces that are identical for the delivery modes, such as age and diet, and their relative abundance by local forces, such as the delivery mode itself. Although the dynamics were similar for specific species, the time of appearance of the C-section associated species seemed to occur earlier than the vaginal delivery-associated species (Supplementary Fig. 16c). To further explore this phenomenon, we examined these two groups of delivery mode-associated species and found that they exhibited significantly different temporal dynamics. This difference was manifested in the relative abundance of specific species as well as at the phylum level (Fig. 4a; see Supplementary Fig. 17 for statistical validation and see Methods: Statistical validation for IndVal-selected species). We observed that these delivery mode-associated microbes appear throughout life, but that the C-section-associated microbes arrive significantly earlier (Fig. 4b, c, *t*-test, *P* < 0.05, two-sided test). The fact that delivery mode-associated microbes were found to arrive continuously from birth until maturity suggests that early-life events have a long-term effect on the rumen microbiome. In other words, animals delivered through C-section and consequently, not inoculated with birth canal microbes, are more prone to invasion. Moreover, we observed that C-section-associated microbes exhibit lower persistence than vaginal delivery-associated OTUs (Fig. 4d, *t*-test, *P* < 0.05, two-sided test), suggesting higher resilience for vaginal delivery-associated species in the rumen environment. Taken together, these findings suggest long-term effects on microbiome composition as a result of different early colonizers induced by different modes of delivery.

### Microbiome temporal dynamics are shaped by early colonizers in the rumen

**Fig. 5:**
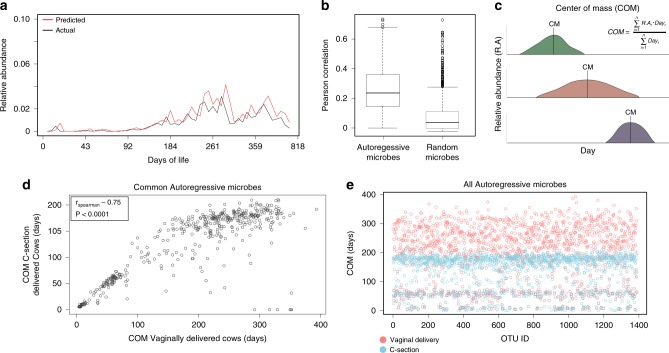
Community dynamics is independent of mode of delivery but there is a time shift in species dynamics. **a** Prediction of a single microbe’s behavior by MTV-LMM method (see Methods section). Black line represents the actual behavior of a given microbe; red line represents predicted behavior. **b** Autoregressive microbes are more predictable than any other randomly selected microbes. Pearson correlation between predicted and actual community composition for autoregressive microbes compared to a random set of microbes. The random set was picked using a permutation test (*n*_permutations_ = 1000) in which at each iteration, 495 microbes are picked and used as predictors for the next time point. **c** Illustration of the center of mass (COM) calculation and clustering. COM is the weighted average for an OTU’s relative abundance over time. $COM = \frac{{\mathop {\sum }\nolimits_{i = 1}^n R.A_t \cdot Day_t}}{{\mathop {\sum }\nolimits_{i = 1}^n Day_t}}$ where i is the cow ID (1–45), *R.An* is the relative abundance of a species at time point *t*, Day_*t*_ is the day of life when the sample was taken (1–831). **d** COM is lower for the C-section-delivered autoregressive microbes. Clustering using the K-means method for the MTV-LMM microbes. Each dot represents the mean COM of autoregressive microbes in a particular cow. Only microbes found to be autoregressive in one mode of delivery, originating either from C-section (blue) or vaginal delivery (red), are presented for each cow. Stars represent the center of the cluster for each delivery mode. Each axis represents the COM values for C-section (*Y*-axis) and vaginal-delivery mode (*X*-axis). **e** Time shift between C-section and vaginal delivery in all autoregressive microbes, with lower COM for microbes from C-section cows. Average COM for all MTV-LMM microbes (OTU index on the *X*-axis, average COM on the *Y*-axis) displayed for either vaginal delivery (red) or C-section (blue), showing a time shift in the COM dependent on the mode of delivery for all OTUs. Each dot represents the COM for a single OTU averaged across all cows within one of the two modes of delivery. Source data is provided as a Source Data file.

Our findings of early life perturbation resulting in altered microbiome composition later in life suggest that a certain microbial composition at a given age or time point could explain the relative abundance of microbial community members at later time points. We therefore sought to understand whether the initial microbiome starting point (mode of delivery) would have an impact on the identity of specific microbiome members. We applied our newly developed tool utilizing a linear mixed model-based method to predict microbial community temporal dynamics^48^ (see Methods: MTV-LMM). Using this method, we quantified the dependence and predictability of the relative abundance of each microbe in our dataset at a given time point, based on the microbial community composition at previous time points (1, .., *t* − 1). We termed these predictable microbes as autoregressive microbes (Fig. 5a). By applying the MTV-LMM method, we detected 1389 autoregressive microbes (Fig. 5b; median Pearson correlation coefficient *r* = 0.26, *P* < 0.0001, *t*-test, two-sided test).

Most of these microbes (91%) belonged to the core successional microbiome and made up 50% of the total identified core successional microbes, suggesting that the large proportion of core successional species depend on historical contingency effects. After identifying the autoregressive microbes, we examined how they are distributed following the two modes of delivery. Our results showed that 401 out of 1389 microbes were autoregressive in both modes of delivery; 494 were autoregressive only in the vaginally delivered animals, and 494 only in the C-section-delivered ones (Supplementary Data 4). We then sought differences in the temporal dynamics of the autoregressive microbes between the two modes of delivery. We found a shift in the temporal dynamics of the autoregressive microbes from the different modes of delivery, as determined by the center of mass (COM) calculation and K-means clustering analysis (Fig. 5d, e; *P* < 0.001, *t*-test, two-sided test; see Methods: COM). Interestingly, we found a high correlation between the COM of the 401 species common to both groups (Spearman correlation, *P* < 0.0001, *r* = 0.75), indicating that the ordering of the OTUs’ COM is conserved between C-section and vaginal delivery, despite the faster dynamics exhibited by the C-section microbiomes. These results, together with our finding of early colonization in the C-section-delivered calves (Fig. 4b), further suggested that early intervention affects the dynamics of microbiome development in C-section animals, showing accelerated microbiome development in terms of both species invasion rate and population dynamics.

Taxonomy analysis showed that the species belonging to the Firmicutes phylum were predominant in autoregressive species found only in C-section delivered cows (Supplementary Fig. 18a, *n* = 494 species). Interestingly, autoregressive species of vaginally delivered cows showed high relative abundance of Proteobacteria until the onset of the more fibrous diet at the age of 90 days (Supplementary Fig. 18b, *n* = 494 species). A rise in relative abundance of the Archaea was recorded in species found to be autoregressive in both groups (Supplementary Fig. 18c, *n* = 401 species).

## Discussion

In the present work, we aimed to elucidate the contribution of historical contingency effects on the assembly dynamics of the rumen microbiome during the first 3 years of life, i.e., from birth to adulthood. Our findings revealed the interplay between deterministic environmental filters controlling the assembly of the rumen microbiome and stochastic factors related to the microbial species pools tapped at early life stages. Deterministic factors, such as age and diet, act globally, affecting microbiome development, independent of mode of delivery. Stochastic factors, in contrast, act locally, i.e., they are specific to individuals or cohorts of individuals with similar life histories.

With respect to global factors, we were able to identify compositional changes in the microbiome that are directly linked to the animal’s age. Thus, we were able to distinguish the effects of animal development from the effects of diet. These included changes in the *Lachnospiraceae* family that changed in relative abundance in a diet-independent manner, or diet-related changes, such as those seen for the methanogenic archaea and *Succinivibrionaceae* family (Fig. 2). Similar to our results, age-related change was also detected in the *Verrucomicrobiaceae* family in sheep, where this microbial family was persistent for over 5 months after birth^49^. Since this bacterial family was previously associated with bovine milk consumption^18,50^, it is reasonable to find it in high relative abundance early in life and to decline when there is an increase in consumption of plant-based feed (Fig. 2d). Interestingly, the *Verrucomicrobiaceae* are linked to maternal transmission of bacteria in mice^51^. It is tempting to speculate that members of this group could be directly affected by the consumption of maternal milk and would play a crucial role in the succession process, like *Bifidobacterium longum*, a resident of the human gut microbiome that is transmitted maternally and directly ferments human maternal milk carbohydrates^52^. Supporting this notion is the finding that *A. muciniphila*, one of the main contributors to the relative abundance of this family, was detected in a study that examined the mouse microbiome in the oral cavity, stomach, small intestine, and large intestine, after administration of breast milk and formula milk^53^. These observations highlight this species as a potential major contributor to the rumen microbiome succession process. To the best of our knowledge, however, this species was not reported to be found in the rumen environment before. Nevertheless, strains of this species were found to be associated with multiple sites in the human body^54^, which suggests that it has a capability for a diverse lifestyle.

Another key global deterministic factor framing the succession process is diet. The increased relative abundance of the *Prevorellaceae* family, in response to fiber-based diet, is in accord with our previous results, in which similar dynamics were described^18,55^. This phenomenon was also described and confirmed by Rey et al.^56^, where an increase in the genus *Prevotella* was detected after transition into solid feed. This bacterial family has a remarkable degree of genetic diversity^57,58^ and occupies various ecological niches within the rumen^5^. The fact that this family was less dominant in the early stages could stem from its tendency to target mostly lignocellulosic polysaccharides^58^, since the newborn calf is fed mostly milk replacer where the *Bacteroidaceae* functions are potentially better fitted^59^.

With respect to local factors, we identified historical contingency effects on many attributes of microbiome assembly and composition, associated with the different initial microbiome pools found in the first 24 h of life following the two modes of delivery (Supplementary Fig. 11). Our analysis revealed that the composition of the microbiome during the first 24 h of life has effects on microbiome structure and dynamics that extend throughout the animal’s life (Fig. 4a). These findings highlight the fact that stochastic events, such as early exposure to different species pools (local effects), act within the framework of the deterministic effects of age and diet (global effects), eventually leading to individual variation. The effects of early intervention in the form of C-section or maternal antibiotic treatment were also reported in humans, where high-level colonization by opportunistic pathogens associated with the hospital environment was detected in babies delivered by Caesarean section^25^. Children born by C-section showed a lack of maternal transmission and significantly less resemblance to their mothers at birth^24,60^. In another study, C-section born babies were also reported to have decreased numbers of the Bacteroidetes phylum^23^.

Interestingly, we found a highly abundant core successional microbiome species that arrived within the first few days of life (Supplementary Fig. 7) and played a key role in assembly dynamics. The core successional microbiome, shared between animals delivered both vaginally and by C-section, explained 37% of the variance and clustering patterns within the microbiome, whereas other microbiome members only explained 8% of the variance (Fig. 3a, PERMANOVA). The importance of this core successional microbiome is in the fact that these microbes are not only shared by the different animals, they are shared between the different time points and persist throughout the entire sampling period, thus the name core successional microbiome. It would be interesting to decipher the mechanism behind their long endurance, as such quality was linked to high strain variability and inter species facilitation^61^ and was yet to be examined in the rumen environment. The fact that core successional members were shared among modes of delivery precludes the explanation that they are acquired via exposure to the maternal birth canal. The source of these core successional microbes thus remains unclear. We hypothesize that some of the core successional species are highly adapted to the rumen environment and present in the environment surrounding the animals, also known as gamma diversity^19^. Unlike the core successional microbiome, the non-core microbiome showed biphasic dynamics: taxa that invaded during the first 140 days were persistent for longer than taxa that arrived later, which turned over at a faster rate (Fig. 3b). The persistence of the early colonizers suggests that, by virtue of arriving early during colonization, early taxa have a competitive advantage over late colonizers. Such an advantage can be mediated by the higher population density of early colonizers and by the modifications they introduce into the environment, which increases the strength of environmental filters^16,62^. These observations reinforce our results showing that early successional species determine the future invasion dynamics of the microbiome. The important contribution of priority effects in this ecosystem allowed us to predict later relative abundance of taxa with high accuracy based on microbiome composition at early time points (Fig. 5b). The fact that these predicted taxa are the most abundant microbiome members and 91% of them are core successional microbes illustrates that historical contingency effects act strongly on the central microbiome members. Interestingly, the order of these species dynamics was similar for the two modes of delivery, while in C-section individuals, they were temporally shifted relative to individuals delivered vaginally (Fig. 5d, e). Furthermore, our analysis showed that for both modes of delivery, microbiome composition at early life stages has a similarly high predictive power for the future dynamics of individual microbes (Supplementary Fig. 19). Taken together, it is suggested that, despite the differences in temporal outcomes between modes of delivery, early events in life determine microbiome development.

The early arrival of C-section-associated microbes suggests that the absence of birth canal exposure and inoculation results in less densely colonized microbiomes that are easier to invade, in line with the higher numbers and appearance rates of C-section-associated species observed in our data (Fig. 4b). Among the delivery mode-associated species, we recorded over 800 C-section-associated species appearing in the rumen ecosystem by 200 days of life, while in vaginally delivered cows only 500 appeared by this time. Interestingly, the autoregressive species dynamics was also different between the delivery mode groups wherein the center of mass of C-section-associated species was significantly lower, by up to 50 percent. The fact that these two non-overlapping microbial groups showed the same trends could be explained by the hypothesis that the environment of C-section-delivered cows is more prone to invasion than vaginally delivered cows. Indeed, recently it was reported that babies born via C-section were more prone to colonization by opportunistic pathogens associated with the hospital environment^25^. Consistent with this notion, the initial species composition acquired through the birth canal contributes to homogenization of microbiome development, relative to C-section individuals whose microbiomes are more variable (Supplementary Fig. 14). Furthermore, we documented higher persistence among species associated with vaginal delivery which could also contribute to this phenomenon (Fig. 4d). In summary, our findings show that both deterministic effects, driven by age and diet, and stochastic effects, driven by early colonization events, shape the composition of the rumen microbiome throughout life. Our findings demonstrate the importance of interventions at early life stages to modulate microbiome development, even under the same husbandry regimes.

## Methods

### Experimental setup

The experiment was conducted at the experimental dairy farm facility of the Volcani Center, Agricultural Research Organization (ARO), Israel, and was approved by the local ethics committee of the Volcani Center (approval number 412/12IL and 566/15IL).

Animal handling and sample collection: Calves (Holstein-Friesian breed) were divided into two groups: those born by C-section (*n* = 18) and those born by vaginal delivery (*n* = 27). Caesarean section is a routine practice for specific breeds of cows^63^, nevertheless, it is not a common practice among most dairy cow breeds. However, in the context of our study, this procedure was used as a disturbance of the colonizing communities created early in life, which enabled us to ask whether it could affect microbiome assembly dynamics throughout the experimental period.

Animals were subjected to conventional housing and growth practices in our experimental facility. Briefly, calves were immediately separated from their mothers after calving to prevent vertical transmission of microbes from the mother’s oral microbiome to their infants. Calves were housed in individual kennels for 90 days. Thereafter, all animals were housed together in corrals. After 90 days, they were transferred to cohabitation groups. These groups were separated according to their diet, age, and sex. Animals were consecutively transferred from one dietary/age group to the next, in order to keep the different groups as homogeneous as possible. Animal metadata, including sex, date of birth, and mode of delivery, are provided in Supplementary Data 5. The overall cohort, 19 males (males were not castrated) and 26 females, was divided into two groups, C-section-delivered calves (*n* = 18) and vaginally delivered calves (*n* = 27). Males were removed from the herd between the ages of 6 and 8 months. Females were sampled for a period spanning 8–28 months. Metadata regarding sample collection can be found in Supplementary Data 6. Females underwent artificial insemination around the age of 480 days and gave birth at around 725 days. Overall, we collected 1634 samples, 1062 samples from vaginally delivered cows, and 573 samples from C-section delivered cows.

Dietary regime: Calves were fed solely colostrum for the first 3 days after calving. From day 4 until 2 months of age (60 days), calves were fed milk replacer and starter mixture ad libitum. After 60 days, calves were weaned and fed only starter mixture until 90 days of age. From day 90 to 180, calves received a low-fiber diet. From day 180 to ~725 days, animals were fed a high-fiber diet. After calving, a low-fiber diet similar to that supplied between 90 and 180 days was provided. All dietary information is supplied in Supplementary Data 7.

Sampling regime: Rumen sampling was carried out using a custom-made stomach tube (Metal Systems, Kiryat Gat, Israel), which was specifically designed for this study with a length of 2500 mm and diameter of 12 mm. This stomach tube was used throughout the entire experiment for all animals. The design of diameter and length of the tube was based on the physiology of pre-ruminant animals and aimed to reach the ventral part of the rumen from birth to adulthood. The manufacturing of the tube included electropolishing, which minimized injuring the esophagus. The sampling protocol was similar to that of Jami et al.^18^ and Shabat et al.^14^, where in younger calves the inserted length of the tube for rumen sampling was based on our initial calibration experiments. In each sampling, the stomach tube was connected to a vacuum pump only when it reached the ventral part of the rumen.

After birth, rumen fluid samples were taken from the newborn calves daily, from day 0 to day 7, due to a previous study in our laboratory that revealed a rapid dynamic changes in microbial composition immediately after birth^18^. Rumen content was sampled twice more between days 7 and 15. After that, rumen content was collected weekly until weaning. Upon weaning on day 60, rumen fluid was collected weekly until at least 220 days of age, after which samples were collected once a month. Experimental setup and dietary regimes are shown in Fig. 1a. Sampling regime for each individual can be found in Supplementary Data 6 and Fig. S2.

Delivery by C-section: C-section was performed according to the protocol approved by the Animal Policy and Welfare Committee of the ARO. Anesthesia was administered to the mothers paravertebrally using lidocaine and adrenaline. The mother was shaved locally, scrubbed with povidone–iodine solution and washed with isopropanol; the C-section was performed using the left-flank laparotomy approach. The mother was then given penicillin and aminoglycosidic antibiotic and recovery was followed until involution of the uterus.

### Bacterial extraction

Thawed rumen samples were transferred to centrifuge bottles and kept on ice for no more than 20 min before processing. Rumen samples were processed as described previously^64^. The samples were centrifuged at 10,000 × *g* and the pellet was dissolved in extraction buffer [100 mM Tris-HCl, 10 mM ethylenediaminetetraacetic acid (EDTA), 3% w/v Tween 80, 0.15 M NaCl, pH 8.0]; 1 g of pellet was dissolved in 4 ml of buffer and incubated at 4 °C for 1 h, as chilling has been shown to maximize the release of particle-associated bacteria from ruminal contents^65^. The suspension was then centrifuged at 500 × *g* for 15 min at 4 °C to remove ruptured plant particles while keeping the bacterial cells in suspension. The supernatant was then passed through four layers of cheesecloth, centrifuged (10,000 × *g*, 25 min, 4 °C) and the pellets were kept at −20 °C until DNA extraction.

### DNA extraction

DNA extraction was performed as previously described^64^. Briefly, cells were lysed by bead disruption with phenol followed by phenol/chloroform DNA extraction. The final supernatant was precipitated with 0.6 volume of isopropanol and resuspended overnight in 50–100 μl TE (10 mM Tris-HCl, 1 mM EDTA), then stored at −20 °C.

### Animal genotyping

Genomic DNA extracts from 36 animals were loaded into a bovine SNP 50 K chip, which is targeted at 54,609 common SNPs that are evenly spaced along the bovine genome (Illumina). The SNP chip model used was Illumina bovine SNP50-24 v3.0, catalog no. 20000766, and it was processed according to the manufacturer’s protocol at the Genomics Center of the Biomedical Core Facility, Technion, Israel.

### Genotype data quality control

QC was performed with the PLINK^66^ program, with the following parameters: -cow—file isgenotype_all—maf 0.05—geno 0.05—mind 0.05—recode12. SNPs that were not genotyped in more than 5% of the individuals were removed. Similarly, individuals were removed from the analysis if they had been genotyped in less than 95% of the loci (SNPs) covered by the SNP chip. Three individuals were removed because of low genotyping, 3001 SNPs were removed because of “missingness” in the genotyped populations, and 15104 SNPs failed the minor allele frequency (MAF) criteria. The total number of SNPs passing QC was 38359.

### Estimating kinship matrix

Cows kinship matrix was built based on autosomal QC-filtered SNP values similarity between cows, by IBS approach using EMMAX^67^ with command line parameters: *emmax-kin-intel64 -v-s-d 10*.

### 16S rRNA gene amplicon sequencing

Sequencing protocols are identical to the earth microbiome protocols see link: http://press.igsb.anl.gov/earthmicrobiome/protocols-and-standards/16s/). Amplification of 16S rRNA gene from the ruminal samples was performed according to Caporaso et al.^68^ for the V4 region, using the primers 515F (5′-GTGCCAGCMGCCGCGGTAA-3′) and 806R (each reverse primer contained a different 12-bp index, see Supplementary Data 8). The protocol was performed under the following conditions: 94 °C for 15 min, followed by 35 cycles of 94 °C for 45 s, 50 °C for 60 s, and 72 °C for 90 s, and a final elongation step at 72 °C for 10 min. The PCR product (380 bp) was cleaned using the DNA Clean & Concentrator™ kit (Zymo Research) and quantified for fragments containing the Illumina adapters. Sequencing was performed using the Illumina Miseq sequencer. For controls in all our runs, we used non-template controls for each of the samples, and therefore all samples were monitored for contamination (see Supplementary Fig. 21 for representative DNA gel pictures). The product was quantified using a standard curve with serial DNA concentrations (0.1–10 nM). Finally, the samples were diluted to a concentration of 4 nM and prepared for sequencing according to the manufacturer’s instructions. The normalized samples were then unified and sequenced by the paired-end method.

### Quality control

Data quality control and analyses were mostly performed using the QIIME 1.9 pipeline^68^. First, after paired ends were joined, reads were demultiplexed, and read-quality filtering was performed using the default settings of the “split_libraries_fastq” command. Total read count for all 1634 samples was 55,493,183 reads, with an average of 33,270 ± 24,875 reads per sample. All samples were then subsampled to 10,000 reads per sample. The next step was to align the obtained sequences to define OTUs for eventual taxonomy assignment. The Uclust method was used to cluster the reads into OTUs using the pick_otus command at 97% similarity. Taxonomy was assigned using the Ribosomal Database Project (RDP) classifier against the 16S Greengenes reference database (http://blog.qiime.org), designated as ‘most recent Greengenes OTUs’. After an OTU table was created, singletons and doubletons were discarded. The OTU table in biom format can be found in Supplementary Data 9.

### Time-point binning

Samples were divided into 58 different time bins according to sampling time; time bins can be viewed in the metadata mapfile (Supplementary Data 6) under the column “time_bins”. Time-point binning was used for α and β diversity over time (Supplementary Figs. 4 and 5) analysis, species arrival rate (Supplementary Fig. 8), and phylum-distribution analysis (Fig. 4a).

### Similarity measurement

For similarity measurement between the bacterial communities in the samples, the Bray–Curtis and UniFrac distance similarity indices were used to compare samples according to both presence and absence of OTUs and relative abundance of OTUs between samples. A PCoA eigenvalues table was calculated using the Bray–Curtis similarity matrix. The beta_diversity.py and principal_coordinates.py Qiime scripts were used to calculate beta-diversity indices. Separation of the different samples within diet clusters was performed using PERMANOVA (qiime script: compare_categories.py). Random Forest classifier was applied using qiime supervised learning.py command.

### Core successional microbes analysis

Core successional microbes were calculated using compute_core_microbiome.py; the OTU table was collapsed by cow id using collapse_samples.py, and then core successional OTUs were calculated as OTUs present in 80% of all cows. Overall, 2544 core OTUs were found. A core heat map was built using the heatmap.2 command in R. OTU table rows (bacterial species, *n* = 2544) were clustered using hclust, and columns (rumen samples, *n* = 1634) were sorted by day of life (see Fig. 3Aii for more details), see Supplementary Data 2 for core microbes OTU table.

### Core successional microbes appearance in different dietary regimes

To examine whether bacterial species tend to be diet-specific, we performed a permutation test (*n* = 100) in which each row was shuffled at each iteration. Thus, the labels comprising each row were changed for each iteration, randomizing time, and diet.

### First appearance of core successional microbes vs. all other microbes

A permutation test was performed in which the mean first presence of the core OTUs was measured. Then 2544 OTUs were randomly selected from a list of all other OTUs (core OTUs excluded) and their first presence was averaged. This step was repeated 1000 times.

### Species arrival rate

A permutation test was performed in which the arrival of new OTUs into each time bin was measured vs. a null model. The null model was created by random shuffling of the time bin labels. This step was repeated 1000 times. The slope was measured for the non-permuted data using a linear regression model (−74) and averaged across all permutations (−134 ± 0.5).

### Persistence analysis

Species persistence was calculated as follows. For each sampling day, we counted the number of species arriving on that day and measured their maximal possible time of appearance within a window of 600 days starting from their day of first appearance (Daylast appearance - Dayfirst appearance). We then averaged the mean time of appearance for each sampling day. This calculation was performed separately on core OTUs and all other OTUs. OTUs appearing later than 430 days of life were discarded due to the lower sampling depth at these time points. We repeated this analysis on OTUs appearing in at least 5, 10, 20, and 30 samples, and received the same results (Supplementary Fig. 9). Nevertheless, we acknowledge the potential confounding effect of ‘low abundant’ molecular species in our analyses, at the level of species that might contribute to the decrease of persistence in the non-core OTUs.

Statistical analysis for persistence of core OTUs vs. all other OTUs was performed as follows: delta (time of last appearance - time of first appearance) was calculated for each OTU, after which sampling days were shuffled and the delta recalculated 100 times. The ratio between real delta and mean permuted delta was calculated. A *t*-test compared the ratio between core OTUs (0.86) and all other OTUs (0.64) and was found to be significant (*P* < 0.005, two sided).

### Unique species at first time point

To test for a significant association between mode of delivery and species appearance on the first day of sampling, we performed a *χ*^2^ test in which the null hypothesis was that there is no relationship between species appearing at time point *t* = 1 and mode of delivery. We used a contingency table in which we had all species arriving at *t* = 1 (*n* = 5067). This table had three columns: the first column was the sum of all species unique to C-section animals (*n* = 1163), the second was the sum of all shared species (*n* = 1665), and the third was the sum for all species unique to vaginally delivered animals (Supplementary Data 10). Our test rejected H0, meaning that there is a dependence between mode of delivery and species arriving at time point *t* = 1 (*P* < 0.05).

### IndVal (delivery mode-associated species)

Because our cohort contained more vaginally delivered than C-section-delivered cows, the first step in performing this analysis was to balance the data. Only 18 vaginally delivered cows were selected, so that the total number of samples from each mode of delivery would be the same. Furthermore, the distribution of the different time points would be as similar as possible. The list of cows selected for each group can be found in Supplementary Data 11.

To determine habitat-associated species (by habitat we refer to C-section or vaginal delivery) among the different communities, we used the R package ‘Labdsv’ and performed the Dufrene–Legendre Indicator Species Analysis (IndVal^47^,), where x = transposed OTU table, clustering = delivery mode, numiter = 1000. Overall, 1041 OTUs were found to be significant for C-section-delivered cows and 809 OTUs were found significant for vaginally delivered cows (i.e., C-section-associated species and vaginal delivery-associated species, accordingly). A list of IndVal-selected species according to mode of delivery can be found in Supplementary Data 3.

### Statistical validation for IndVal-selected species

Since these habitat-associated species accounted for <2% of the overall species cohort, we sought a validation method to determine the significance of these species along the entire sample cohort. We counted the number of habitat-associated species (OTUs) with C-section and vaginal delivery as the differentiating ecological identity and compared this to 100 permutations in which the delivery-mode labels were shuffled (Supplementary Fig. 15).

### Phylum-distribution analysis

The distribution of the four main phyla (Actinobacteria, Bacteroidetes, Firmicutes, and Proteobacteria) was calculated by counting the number of OTUs belonging to each of these phyla within each time bin. The difference between the distribution of each phylum between the two modes of delivery was determined by comparing the COM between the two modes of delivery by randomly sampling 70% of the values independently for C-section and vaginal delivery and calculating COM (*n* = 1000 times). We then compared the two vectors for each phylum (1000 COM values for C-section and 1000 COM values for vaginal delivery) using Wilcoxon test (Supplementary Fig. 17).

The kernel density plot was used to present the distribution of the data (Fig. 3a). Kernel density is a weighting function that quantifies the density of samples and presents them in a smooth manner^69^. We used the kernel density in order to present a histogram of the density of different phyla along time. Kernel smoothing estimates were applied to each subpopulation (C-section and vaginal delivery) and presented only the four main phyla (Actinobacteria, Bacteroidetes, Firmicutes, and proteobacteria). In Kernel density, Areas with greater point density, in this case higher density of specific phylum, will have higher kernel estimate values at a specific time point, as can be seen in Fig. 3a.

### COM

COM is the average time of appearance for an OTU, weighted according to its relative abundance across all sampling points. COM was calculated for each OTU as:

$${\mathrm{COM}} = \frac{{\mathop {\sum }\nolimits_{i = 1}^n R.A_t \cdot {\mathrm{Day}}_t}}{{\mathop {\sum }\nolimits_{i = 1}^n {\mathrm{Day}}_t}}$$

where *i* is the cow ID (1–45), *R.An* is the relative abundance of a species at time point *t*, Day_*t*_ is the day of life when the sample was taken (1–831).

### MTV-LMM

MTV-LMM uses a linear mixed model for identifying autoregressive taxa and predictioning their relative abundance at future time points (see Shenhav et al.^48^ for more details). MTV-LMM is motivated by our assumption that the temporal changes in the relative abundance of taxa j are a time-homogeneous high-order Markov process. MTV-LMM models the transitions of this Markov process by fitting a sequential linear mixed model (LMM) to predict the relative abundance of taxa at a given time point, given the microbial community composition at previous time points. Intuitively, the linear mixed model correlates the similarity between the microbial community composition across different time points with the similarity of the taxa relative abundance at the next time points. MTV-LMM is making use of two types of input data: (1) continuous relative abundance of focal taxa j at previous time points and (2) quantile-binned relative abundance of the rest of the microbial community at previous time points. The output of MTV-LMM is prediction of continuous relative abundance, for each taxon, at future time points.

In order to apply linear mixed models, MTV-LMM generates a temporal kinship matrix, which represents the similarity between every pair of samples across time, where a sample is a normalization of taxa relative abundances at a given time point for a given individual. When predicting the relative abundance of taxa j at time *t*, the model uses both the global state of the entire microbial community in the last q time points, as well as the relative abundance of taxa j in the previous *p* time points. The parameters *p* and *q* are determined by the user, or can be determined using a cross-validation approach; a more formal description of their role is provided in the Methods. MTV-LMM has the advantage of increased power due to a low number of parameters coupled with an inherent regularization mechanism, similar in essence to the widely used ridge regularization, which provides a natural interpretation of the model.

### Training and testing the model

We divide our dataset into three parts: training, validation, and testing, where each part is ~1/3 of the time series (sequentially). We train all four models presented above and use the validation set to select a model for each taxon j, based on the highest correlation with the real relative abundance. Using the validation set, we found *p* = 1 and *q* = 1 to be the best model for most taxa and therefore used these parameters. We then compute sequential out-of-sample predictions on the test set with the selected model.

### Quality control for sequencing errors

We measured the probability of each OTU in our data set to arise from sequencing errors. Using a method by Jing et al.^70^ we calculated Poisson probabilities for a single sequence at different similarities for each OTU, considering sequencing error rate of 0.24% as measured for illumina amplicon sequencing^71^. We defined minor and major OTUs as described in the above method and assessed whether they are artifacts arising from sequencing errors. The probability of each minor OTU arising by sequencing error was determined by multiplying this probability with the number of nucleotides in a given biological sample (which in our case is 250 nucleotides). We next tested for random sequencing error hypothesis for each ‘major’ and ‘minor’ OTUs-species (97% clusters). More than 92% and 94% respectively of the OTUs rejected the null hypothesis (using Benjamini Hochberg multiple hypotheses adjustment). Supplementary Data 12 describes in detail the probability of each minor OTU to represent sequencing errors and Supplementary Data 13 which supplies the OTUs that were not found to be significant after Bonferroni correction.

Finally, we sequenced several technical replicates of several samples in order to detect and calculate the number of spurious OTUs and the error rate. These analyses revealed that the technical replicates are highly similar to each other and contain very low amount of variance compared to biological samples which is mainly due to sampling of rare OTUs as previously described (Supplementary Fig. 20)

We additionaly examined the probability of the non core OTUs presented in Fig. 2b as gray dots (non-core OTUs) to result from sequensing errors. Using the analysis mentioned above we calculated the probability of these OTUs to be the result of sequencing error and found them all to be genuine and to reject the null hypothesis. In addition to this analysis we have measured the robustness of our results by gradually increasing the stringency of our analysis and measuring the validity and robustness of our findings. This analysis resulted in the same conclusions, further strengthening the robustness of these findings. We examined OTUs that appeared in {5, 10, 20, 30} samples (Fig S9) which decrease the probability of these OTUs to be a sequencing error to a maximum of 7.96 × 10^−14^. This is calculated in the following manner: The error rate per nucleotide that we used is 0.0024 (0.24%). If a given sequence belongs to some focal 97% cluster (let it be cluster A) and was erroneously classified to cluster B, it should have at least eight different nucleotides compared to the OTUs in cluster A. In the most stringent settings, that would be due to an error in one nucleotide, with a probability of 0.0024. Our results show that this stringent analysis did not affect our findings of both core and non-core persistence. With regard to the autoregressive OTUs, we only used OTUs with at least 10% prevalence (appear in at least 160 samples) and for which the probability of being spurious is very low (0.0024^16^).

### Quality control by sequencing synthetic communities

In order to test the occurrence of sporius taxa during our clustering and taxonomy assignment processes, we have created triplicates of synthetic consortia composed of 2 and 3, 4, and 5 microbes (See table in Supplementary Fig. S23). We sequenced and analyzed these consortia using the same parameters as described above. Our results accurately identified the species that were introduced into the consortia. Nevertheless, they also show the occurrence of sequence errors that were on average only a negligible fraction (0.0002) of the total relative abundance (Supplementary Fig. 23). We acknowledge the potential confounding effect of ‘low abundant’ molecular species.

### Reporting summary

Further information on research design is available in the Nature Research Reporting Summary linked to this article.

## Supplementary information

Supplementary Information

Description of Additional Supplementary Files

Supplementary Data 1

Supplementary Data 2

Supplementary Data 3

Supplementary Data 4

Supplementary Data 5

Supplementary Data 6

Supplementary Data 7

Supplementary Data 8

Supplementary Data 9

Supplementary Data 10

Supplementary Data 11

Supplementary Data 12

Supplementary Data 13

Reporting Summary

## Data Availability

All sequencing files and metadata are deposited in SRA under PRJNA591750. The source data underlying Figs. 1b, 2a–e, 3a, b, 4a–d, and 5a, b, d, e, and Supplementary Figs. 1–20 and 23 are provided as a Source Data file.

## Source Data

Source Data
