# Supplementary material for: Stochasticity constrained by deterministic effects of diet and age drive rumen microbiome assembly dynamics
Source: Nat Commun. 2020 Apr 20;11:1904. doi: 10.1038/s41467-020-15652-8 (PMC7170844; doi:10.1038/s41467-020-15652-8)
Supplement: Supplementary file 1 — Supplementary Information [file 41467_2020_15652_MOESM1_ESM.pdf]

Stochasticity constrained by deterministic effects of diet and age drive rumen microbiome assembly dynamics

Ori Furman[1#], Liat Shenhav[2#], Goor Sasson[1], Fotini Kokou[1], Hen Honig[3], Shamay Jacoby[3], Tomer Hertz[4], Otto.X Cordero[5], Eran Halperin[2], Itzhak Mizrahi[1*]

[1]Department of Life Sciences, Ben-Gurion University of the Negev and the National Institute for Biotechnology in the Negev, Marcus Family Campus, Beer-Sheva, Israel

[2]Department of Computer Science, University of California Los Angeles, Los Angeles, CA, USA

[3]Institute of Animal Sciences, Agricultural Research Organization, Rishon Letziyon, Israel

[4]The Shraga Segal Department of Microbiology, Immunology and Genetics, Ben-Gurion University of the Negev and the National Institute for Biotechnology in the Negev, Marcus Family Campus, Beer-Sheva, Israel

[5]Department of Civil and Environmental Engineering, Massachusetts Institute of Technology, Cambridge, MA 02139, USA

[#]These authors contributed equally

[*]Corresponding author: Prof. Itzhak Mizrahi, Department of Life Sciences, Ben-Gurion University of the Negev, Marcus Family Campus, Beer-Sheva, Israel. Email: imizrahi@bgu.ac.il; Tel. (+972) 8 647 9836.

Supplementary Figures

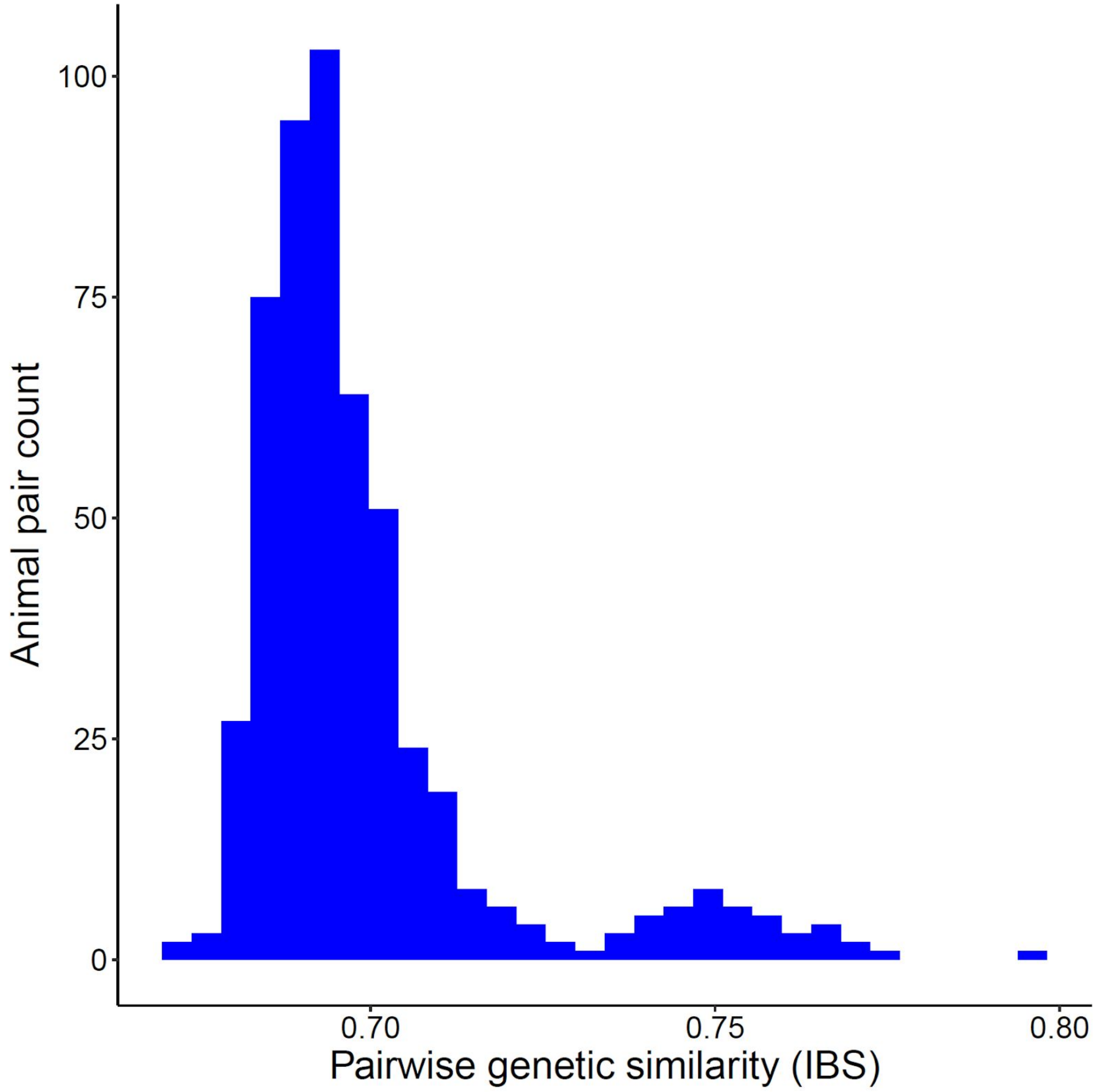

Supplementary Figure 1 - **Pairwise host genetic similarity based on 33 out of 45 animals**. No evidence was found of host genetic similarity within delivery-mode groups (PERMANOVA, $P = 0.316$). In addition, host genetics did not contribute to the similarity in rumen microbial composition and relative abundance (Mantel test, $P = 0.788$). Source data is provided as a Source Data file.

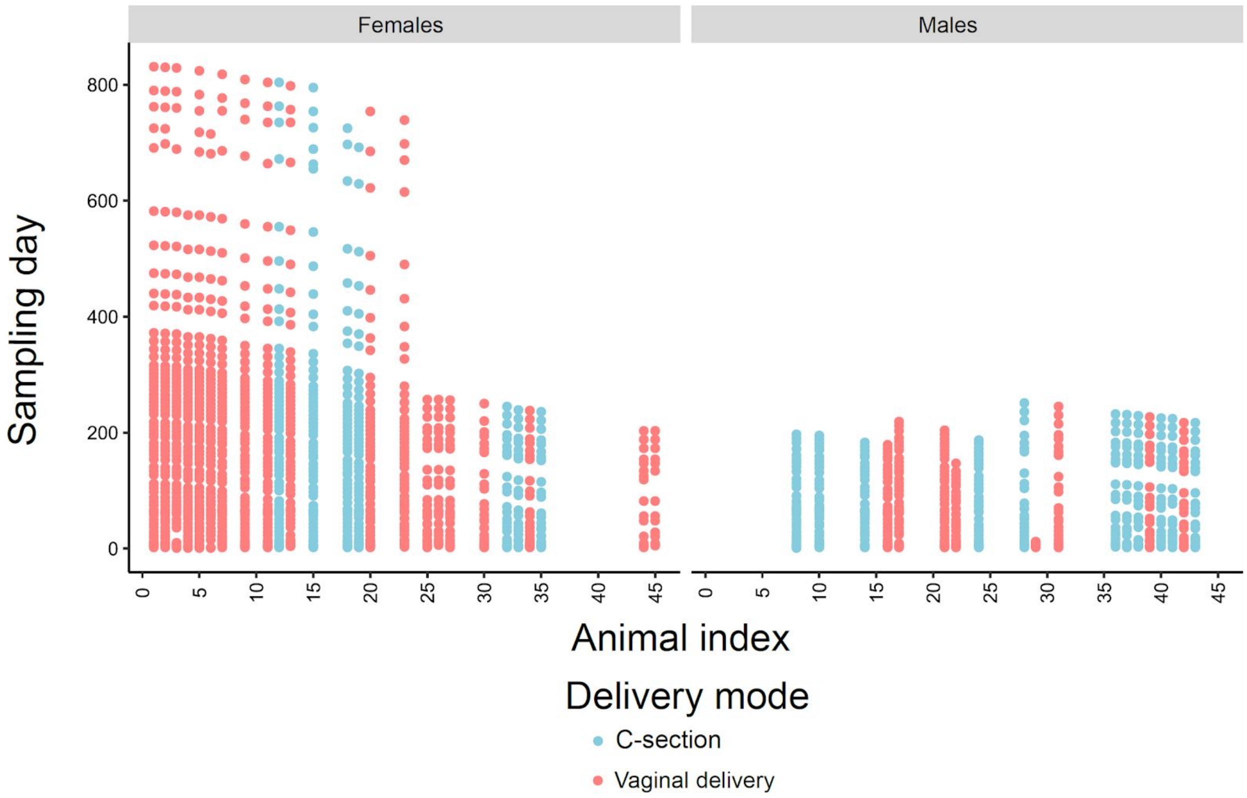

Supplementary Figure 2 - Sampling regime for all animals. Animals were divided between females (left panel) and males (right panel). X-axis represents animal index. Y-axis represents the number of samples (n = 1634 samples across all animals) and sampling days. Blue dots are C-section-born animals (n = 18 animals) and red dots are vaginally delivered animals (n = 27 animals). For further elaboration, see Supplementary Data 6. Source data is provided as a Source Data file.

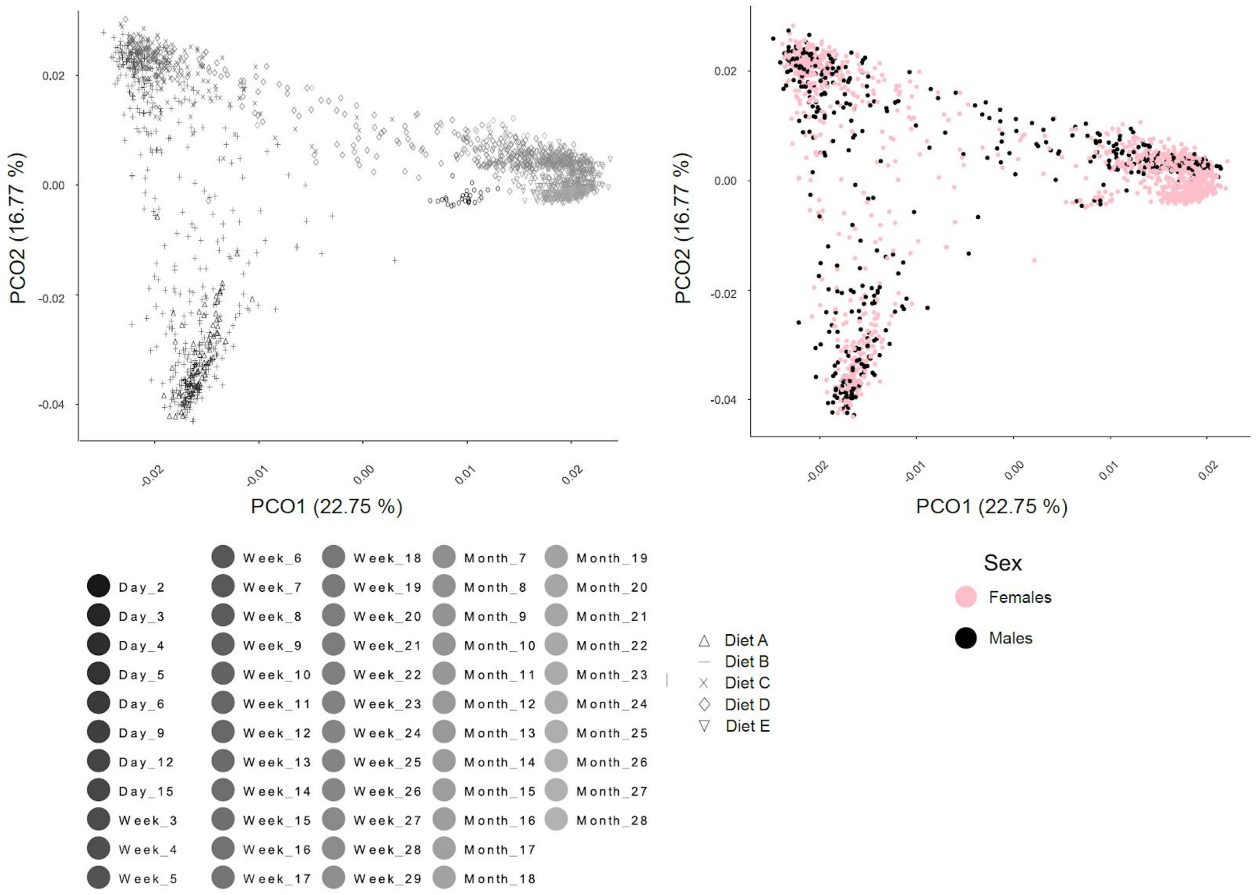

Supplementary Figure 3 - Dynamics of the different microbial families is shaped by age and diet. A. Principal coordinate analysis (PCoA), based on Bray–Curtis metrics, showed clustering of operational taxonomic units (OTUs) according to age and diet (PERMANOVA, P = 0.001). Animals were fed five different diets during the sampling period. Each dot represents a single sample (n = 1634). Samples are colored in grayscale, where darker colors represent older ages. Shapes of the different samples represent the different diets. **B.** Principal coordinate analysis (PCoA), based on Bray–Curtis metrics, showed clustering of operational taxonomic units (OTUs), according to sex. Each dot represents a single sample (n = 1634). Samples are colored according to animal sex, which was not found to be a significant factor for clustering of the samples. Females are colored in red and males in black. Source data is provided as a Source Data file.

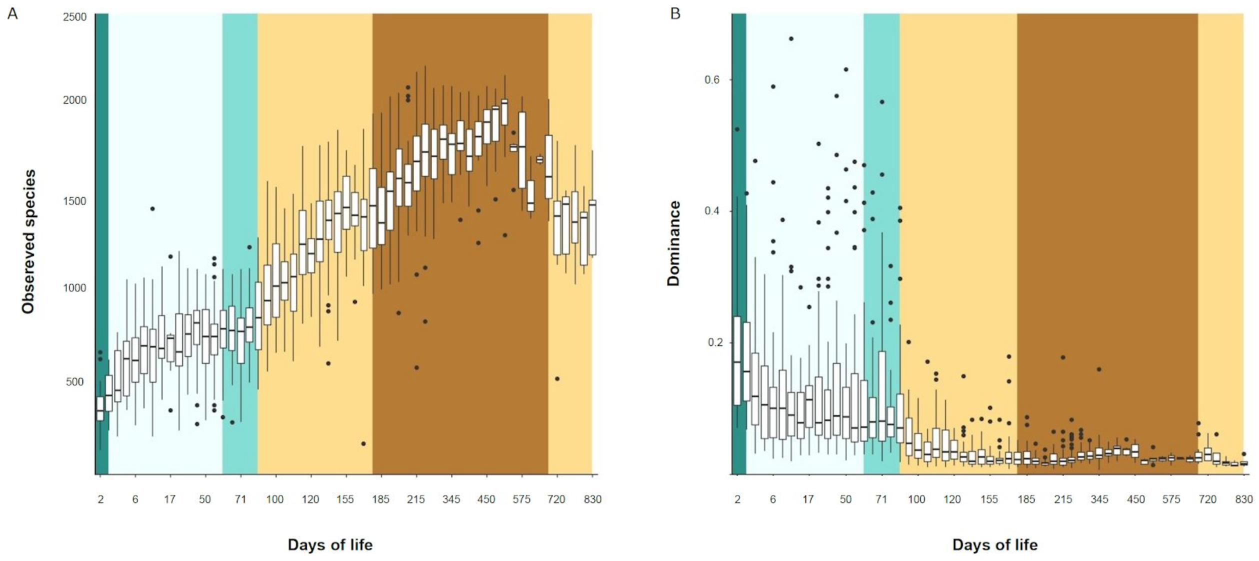

Supplementary Figure 4 - Sample diversity increases over diet and age. A. Number of observed species increased over age and diet. **B.** Dominance of different samples across time, Y-axis represents the number of observed species. X-axis represents age. Samples (n = 1634) were binned according to age (n = 58 time bins); time bins are defined in Supplementary Data6. Boxes represent the interquartile range (IQR) between the first and third quartiles (25th and 75th percentiles, respectively) and the horizontal line inside the box defines the median. Whiskers represent the lowest and highest values within 1.5 times the IQR from the first and third quartiles, respectively. Source data is provided as a Source Data file.

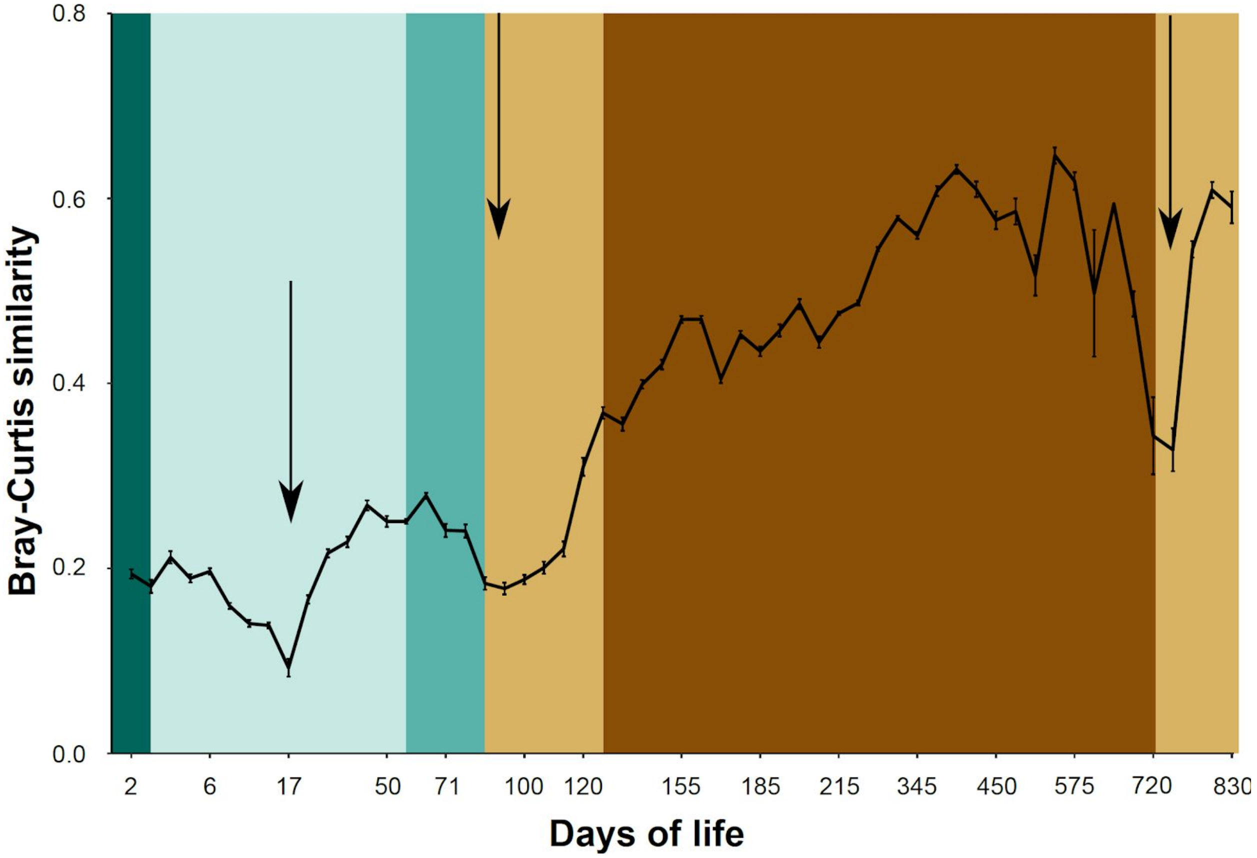

Supplementary Figure 5 - **Similarity (Bray–Curtis) within each time bin increases across time with periodical decreases during dietary changes**. Y-axis represents similarity in microbiome composition using the Bray–Curtis measure of microbial species relative abundance. Error bars represent standard error of mean, samples (n = 1634) were binned according to age (n = 58 time bins); time bins are defined in Supplementary Data 6. Source data is provided as a Source Data file.

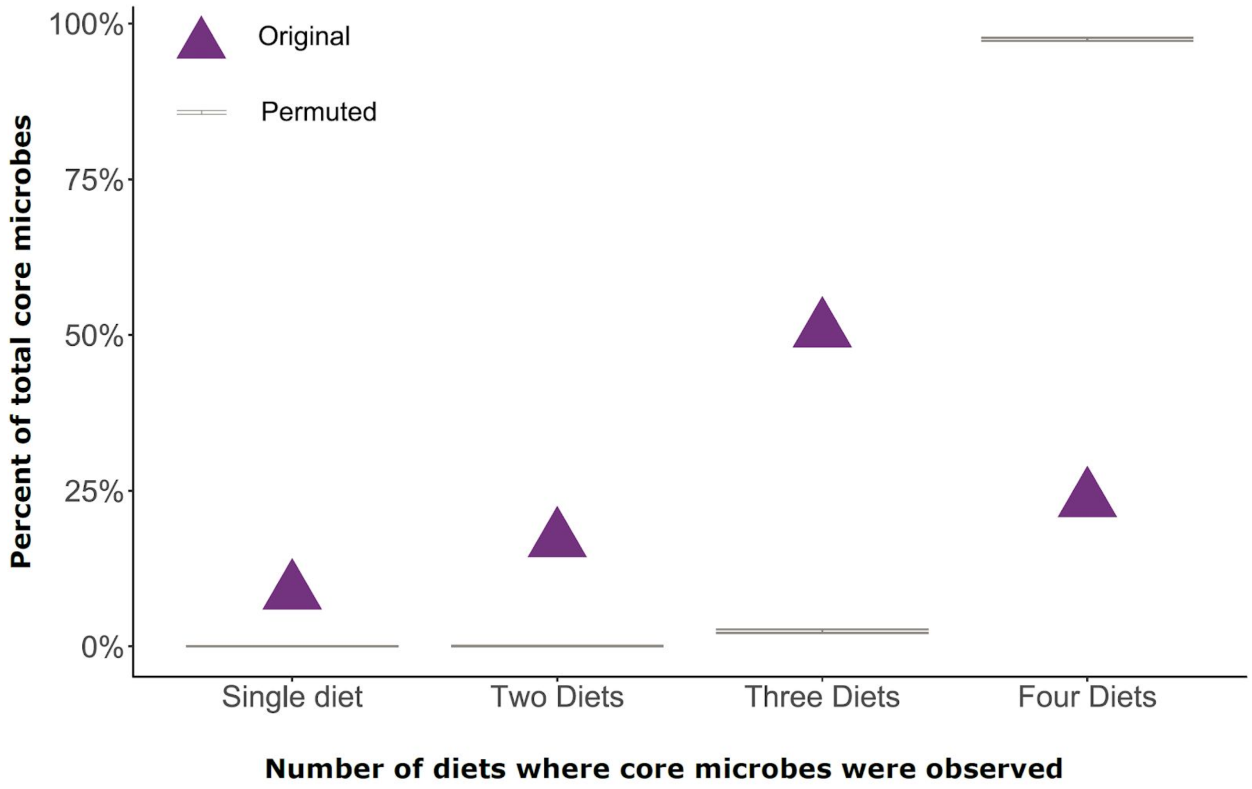

Supplementary Figure 6 - **Core successional microbe appearance under different dietary regimes.** To examine whether bacterial species tend to be diet-specific, we performed a permutation test (n = 100), in which each row was shuffled at each iteration. Thus, the labels comprising each row were changed for each iteration, randomizing time and diet. Source data is provided as a Source Data file.

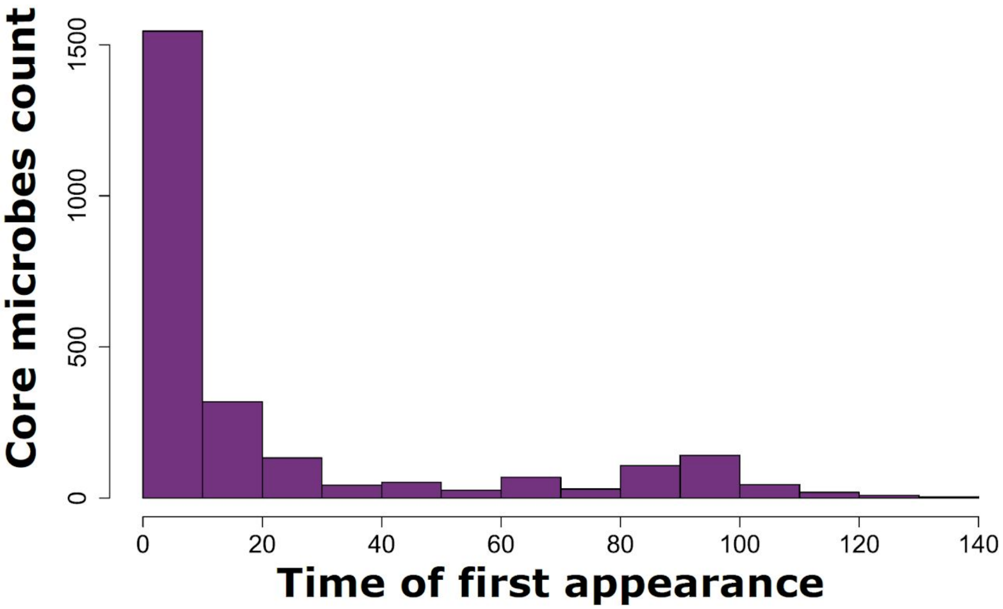

Supplementary Figure 7 - **Most core successional species appear as early as the first week of life**. A histogram describing time of first appearance of core successional microbes (n = 2544 OTUs) in the host. Source data is provided as a Source Data file.

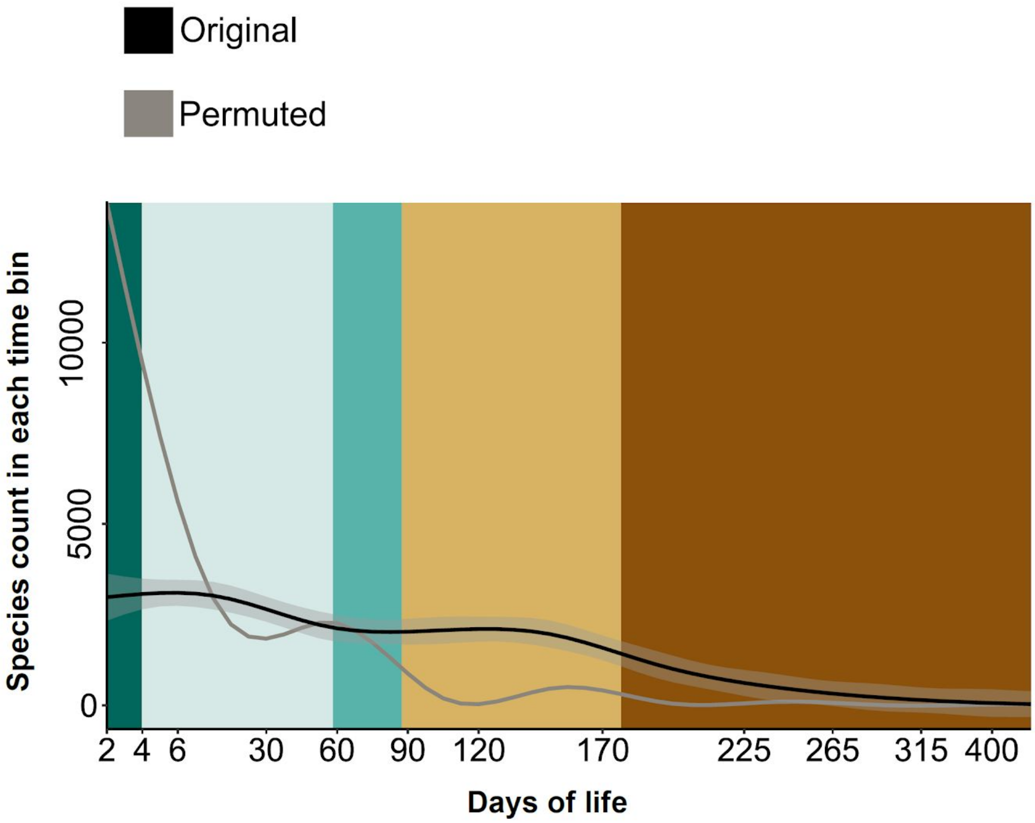

Supplementary Figure 8 - **Time is a prominent factor in the appearance of new species in the host.** Smoothed curve plot describing the number of new microbial species appearing in the rumen as a function of age (the earliest time bin in which it was observed in any given animal (see Methods: Species arrival rate). Time bins are defined in Supplementary Data 6. Source data is provided as a Source Data file.

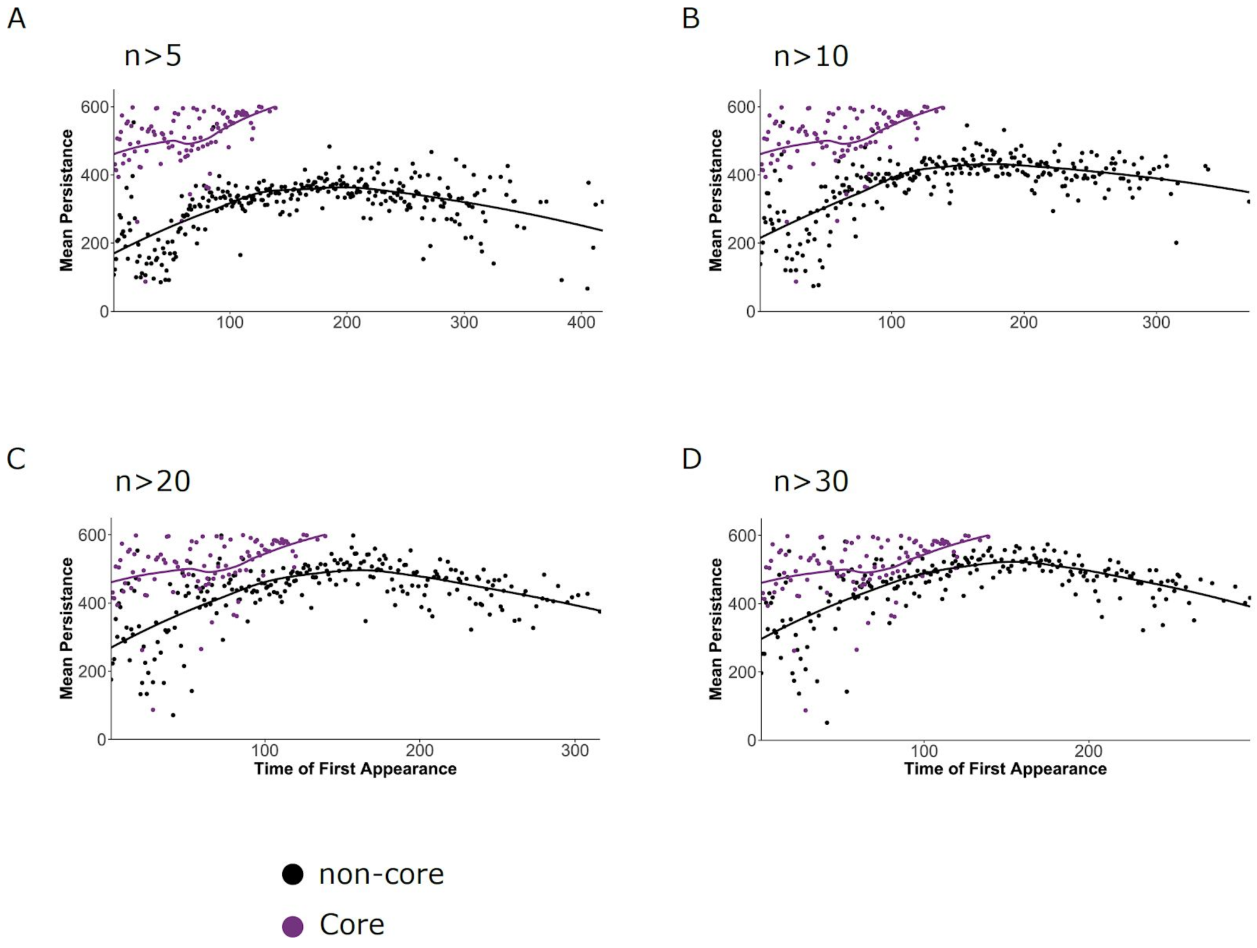

Supplementary Figure 9 **- Non-core taxa are less persistent than the core successional microbiome.** Core successional species persistence (Y-axis) as a function of time of appearance (X-axis). Non-core species were screened by the minimum number of samples in which they were detected: **A.** Minimum of 5 samples (n = 43423 species). **B.** Minimum of 10 samples (n = 24533 species). **C.** Minimum of 20 samples (n = 13648 species). **D.** Minimum of 30 samples (n = 9683 species). Each dot represents the average persistence of all microbes that appeared in the ecosystem on the specified day. Species appearance was measured over a 600-day window from first appearance, and persistence was calculated as the mean of the $\Delta(t_{first\ appearance} - t_{last\ appearance})$. Purple dots represent core successional microbes, grey dots represent non-core microbes. Source data is provided as a Source Data file.

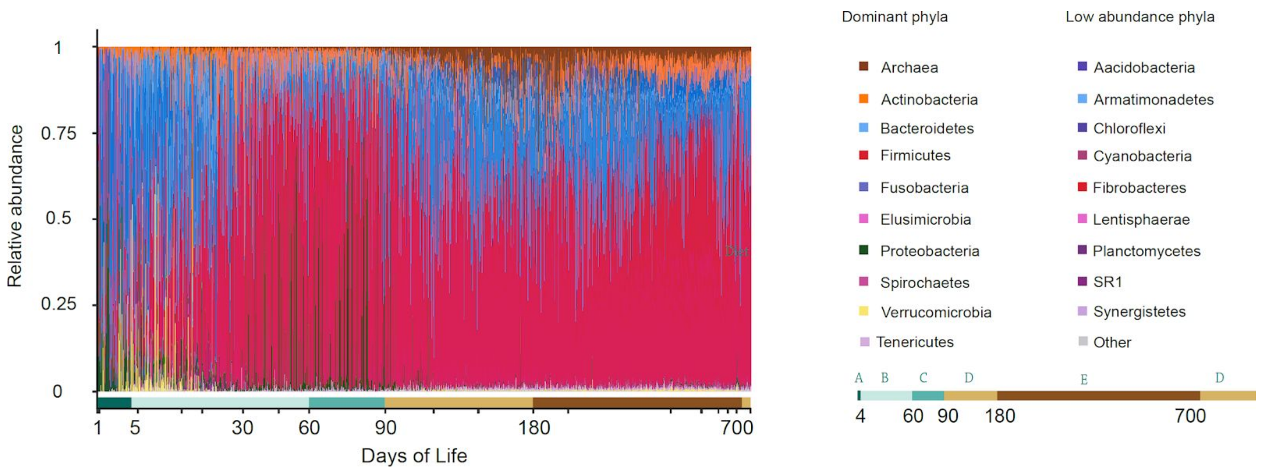

Supplementary Figure 10 - **The core successional microbiome has similar dynamics to the overall microbiome. A.** Relative abundance of the 91 microbial families for the 2544 species found to be present in at least 80% of the animals. Relative abundance values were re-calculated using these species only. Y-axis represents the re-calculated relative abundance, X-axis represents all samples (n = 1634), sorted by sampling day. All families belonging to the same phylum are colored by different shades of the same color. The main phyla are described on the right of the figure**,** see Supplementary Figure 22 for the full family level color code. Source data is provided as a Source Data file.

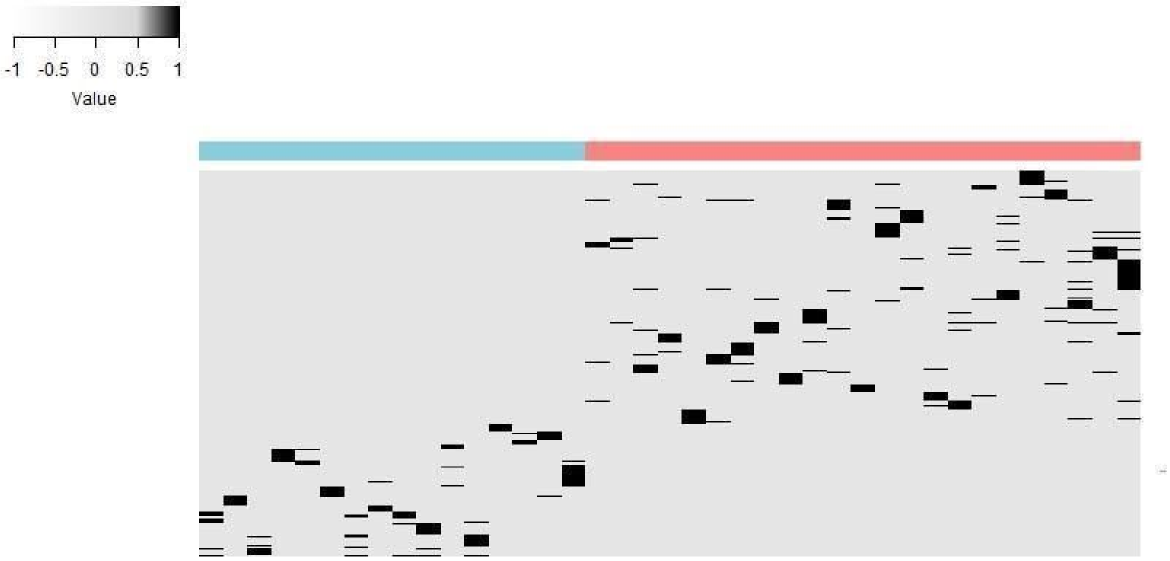

Supplementary Figure 11 - **Heat map showing species unique for each mode-of-delivery group at the first time point**. Each row represents a different OTU, each column represents the first time point for C-section animals (blue) and vaginally delivered animals (red). The Chi square test found significant dependence between mode of delivery and species appearance at time point t = 1 (*P* < 0.05). Source data is provided as a Source Data file.

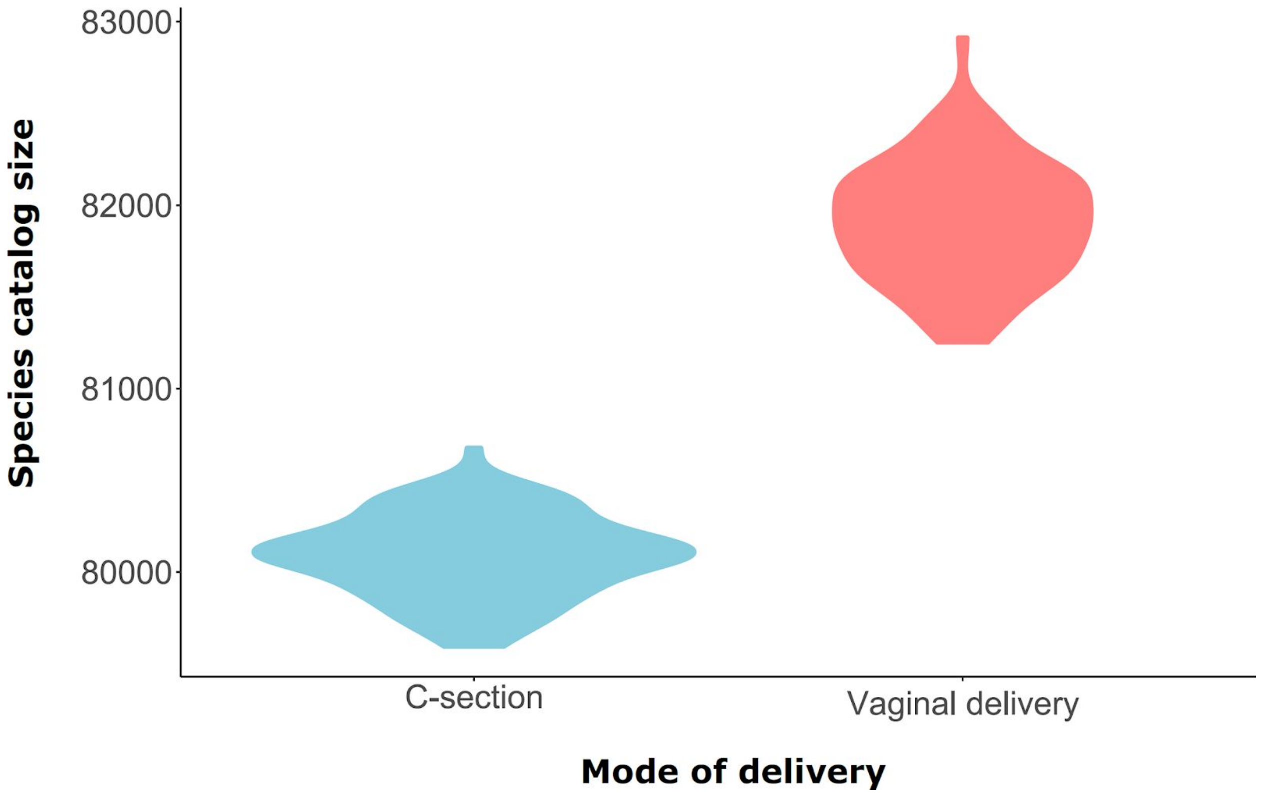

Supplementary Figure 12 - **Vaginally delivered animal sub cohort shows a richer species catalog**. Species catalog per mode of delivery, showing a higher number of species originating from the vaginal vs. C-section delivery mode. A bootstrap approach was taken, where 500 animal microbiome samples from each delivery (total 1000) were randomly picked, and species catalog size was calculated. The procedure was repeated 1000 times, and species catalog sizes were compared between delivery modes (Wilcoxon test, P = 2.2*10^{-16}< 0.0001, two sided test). Source data is provided as a Source Data file.

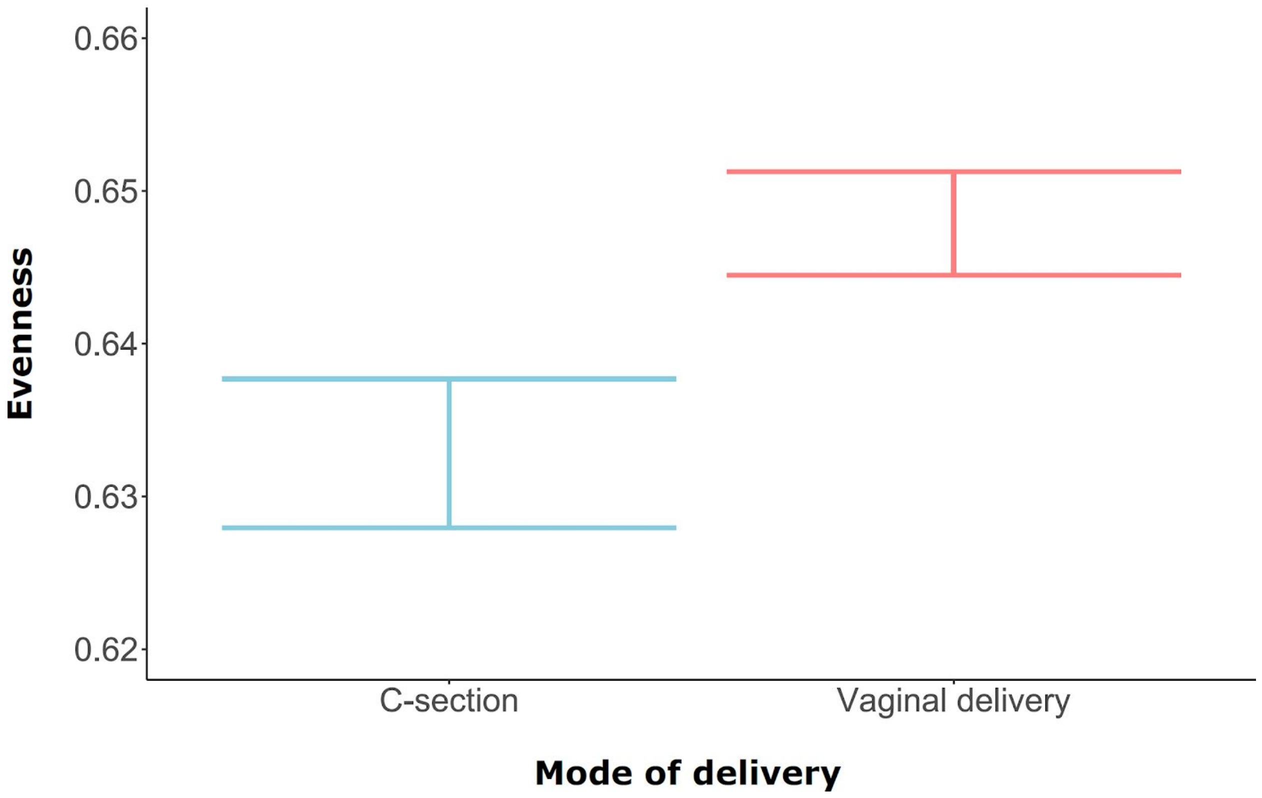

Supplementary Figure 13 - **Evenness per mode of delivery, showing higher values in vaginally delivered cows compared to C-section-delivered cows** (Wilcoxon test, *P* = 0.0135, two sided test). Source data is provided as a Source Data file.

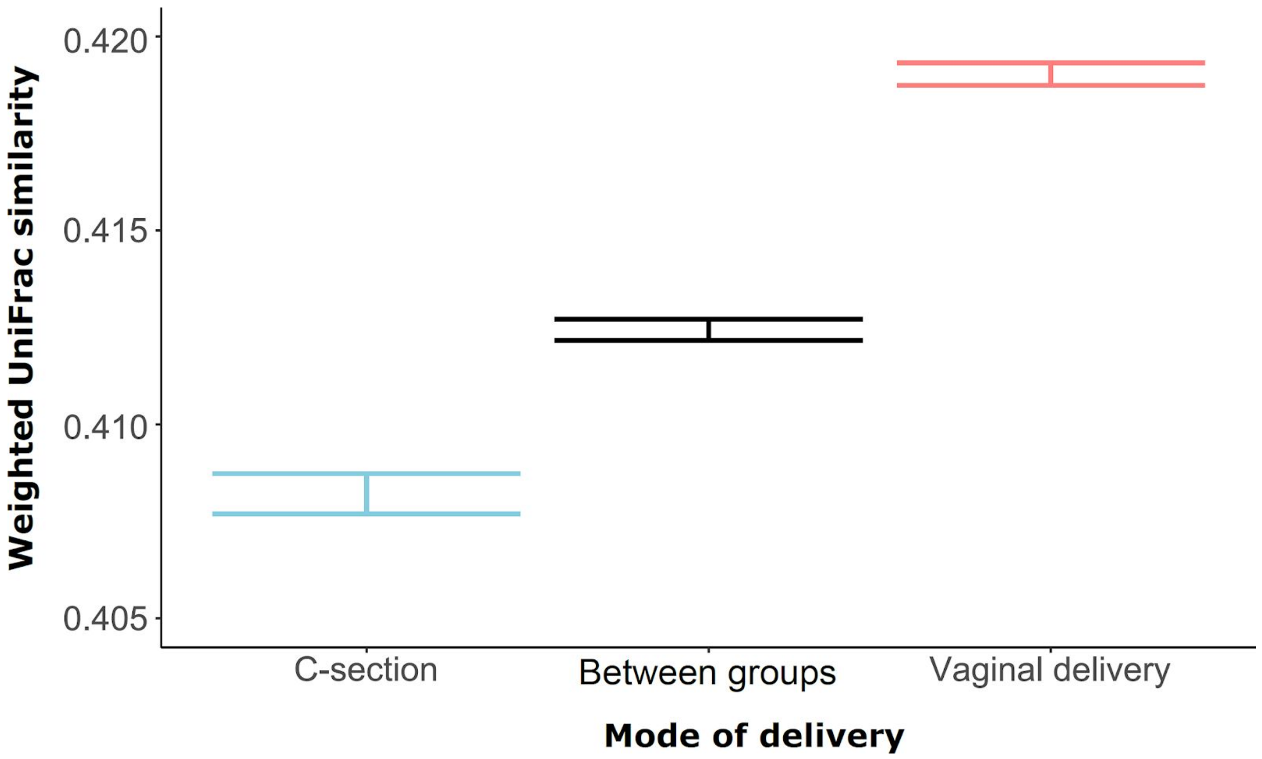

Supplementary Figure 14 - **Weighted UniFrac similarity, calculated within and between delivery-mode groups**. Higher similarity was found in the vaginally delivered cows (non-parametric Bonferroni-corrected, using 1000 Monte Carlo simulations, P = 0.01). Source data is provided as a Source Data file.

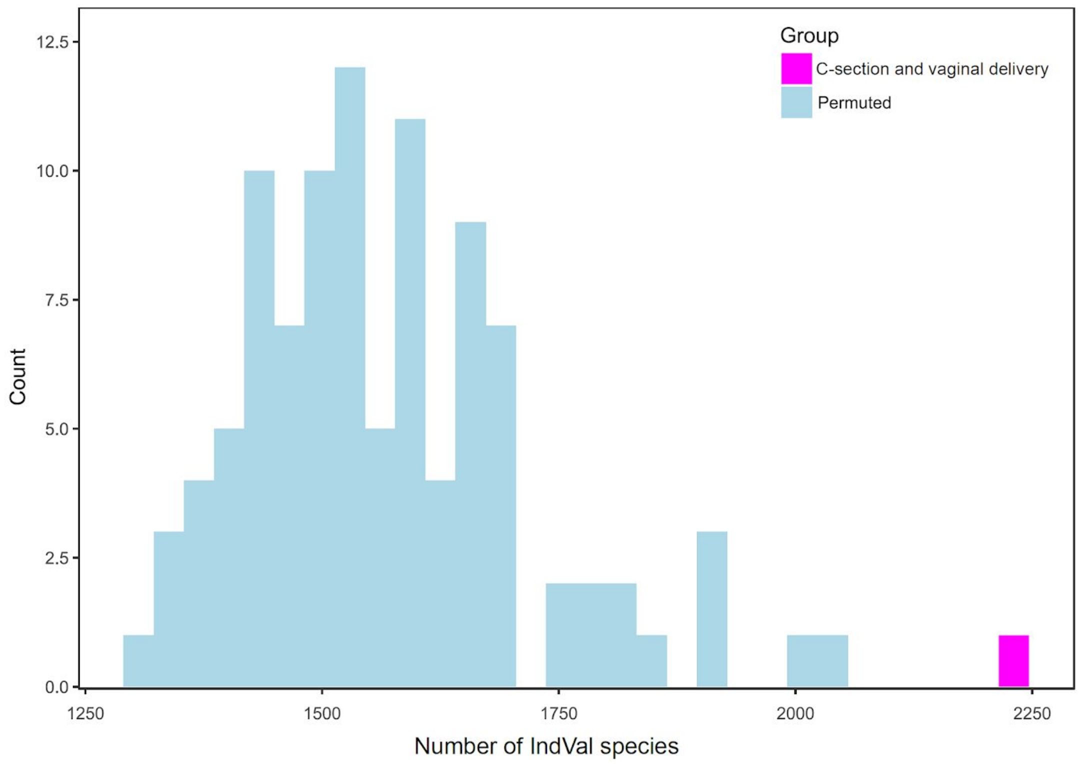

Supplementary Figure 15 - **Statistical validation for IndVal-selected species.** Number of habitat-associated species (OTUs) for C-section and vaginal delivery as the differentiating ecological identity, compared to 100 permutations where the delivery-mode labels were shuffled. The test was performed on 18 animals in each group, where nine vaginally delivered cows were omitted. Source data is provided as a Source Data file.

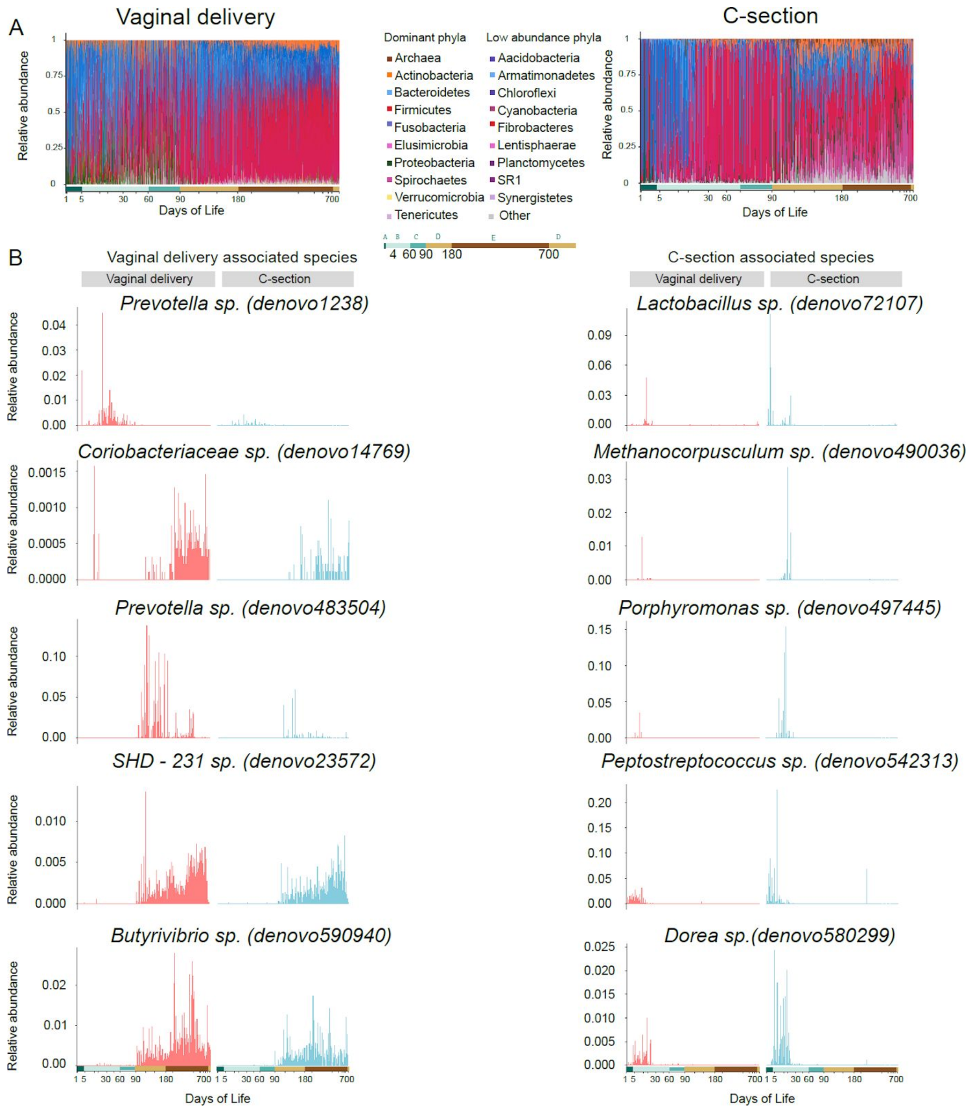

Supplementary Figure 16 - **Microbial dynamics are altered by mode of delivery. A.** Relative abundance of delivery mode-associated species (values were re-calculated using these species only). Left panel, relative abundance of the 81 microbial families of the 809 species found to be associated with vaginally delivered animals. Right panel, relative abundance of the 59 microbial families for the 1041 species found to be associated with C-section animals. Y-axis represents the recalculated relative abundance, X-axis represents all of the respective samples, sorted by sampling day. All families belonging to the same phylum are colored by different shades of the same color, the main phyla are described at the bottom of the figure. See Supplementary Figure 22 for the full family level color code. **B.** Relative abundance of 10 representative microbial species, generally associated with either vaginal delivery (Left panels) or C-section (Right panels). Each panel is divided into the relative abundance of the specific species in the relevant delivery mode. Y-axis, values were calculated based on the relative abundance of all microbial species. Source data is provided as a Source Data file.

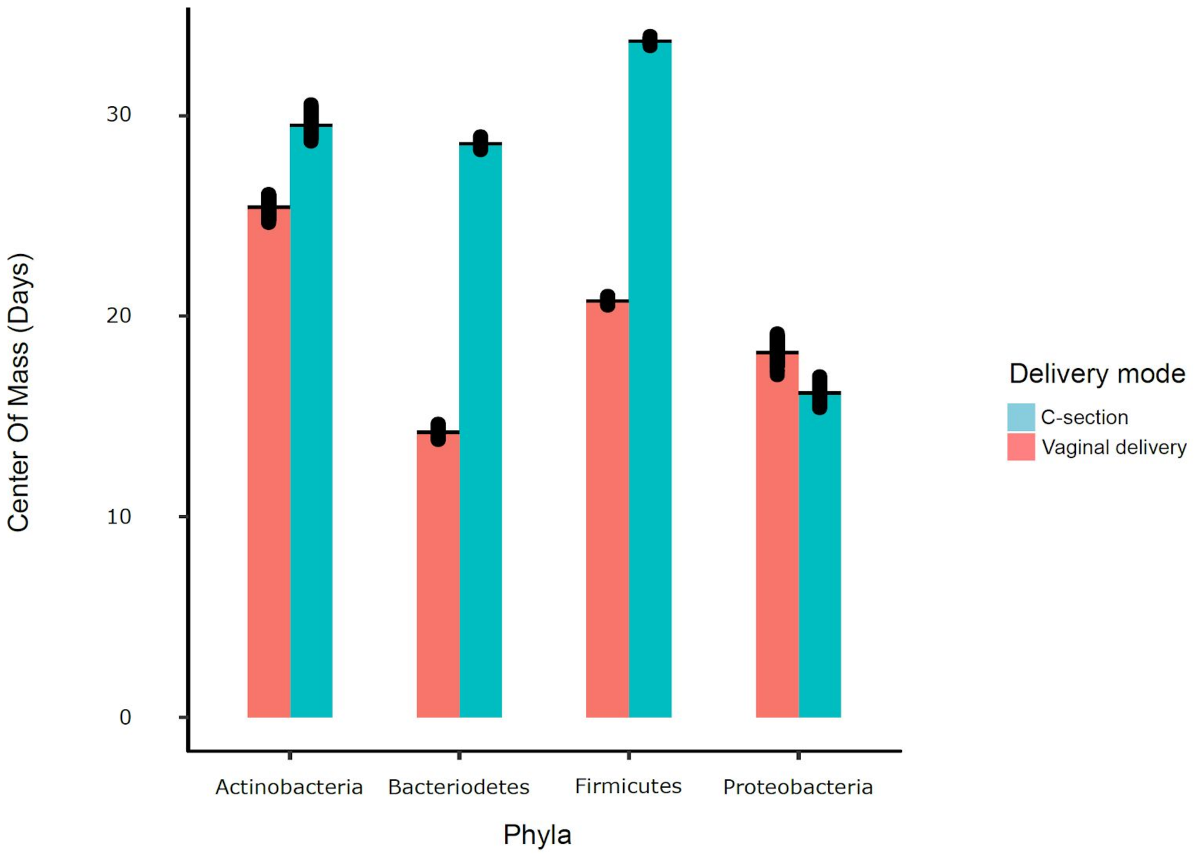

Supplementary Figure 17 - **Statistical validation for phylum-distribution analysis.** The distribution of the four main phyla (Actinobacteria, Bacteroidetes, Firmicutes and Proteobacteria) was calculated by counting the number of OTUs in each of these phyla within each time bin. The difference between the distribution of each phylum between the two modes of delivery was determined by comparing the Center Of Mass COM (see Methods: COM) between the two modes of delivery by random sampling of 70% of values, independently for C-section and vaginally delivered cows, and calculating COM (n = 1000 times). We then compared the two vectors for each phylum (1,000 COM values for C and 1,000 COM values for N) using Wilcoxon test. Error bars (horizontal lines on the top of each bar) represent the calculated standard error of mean. Source data is provided as a Source Data file.

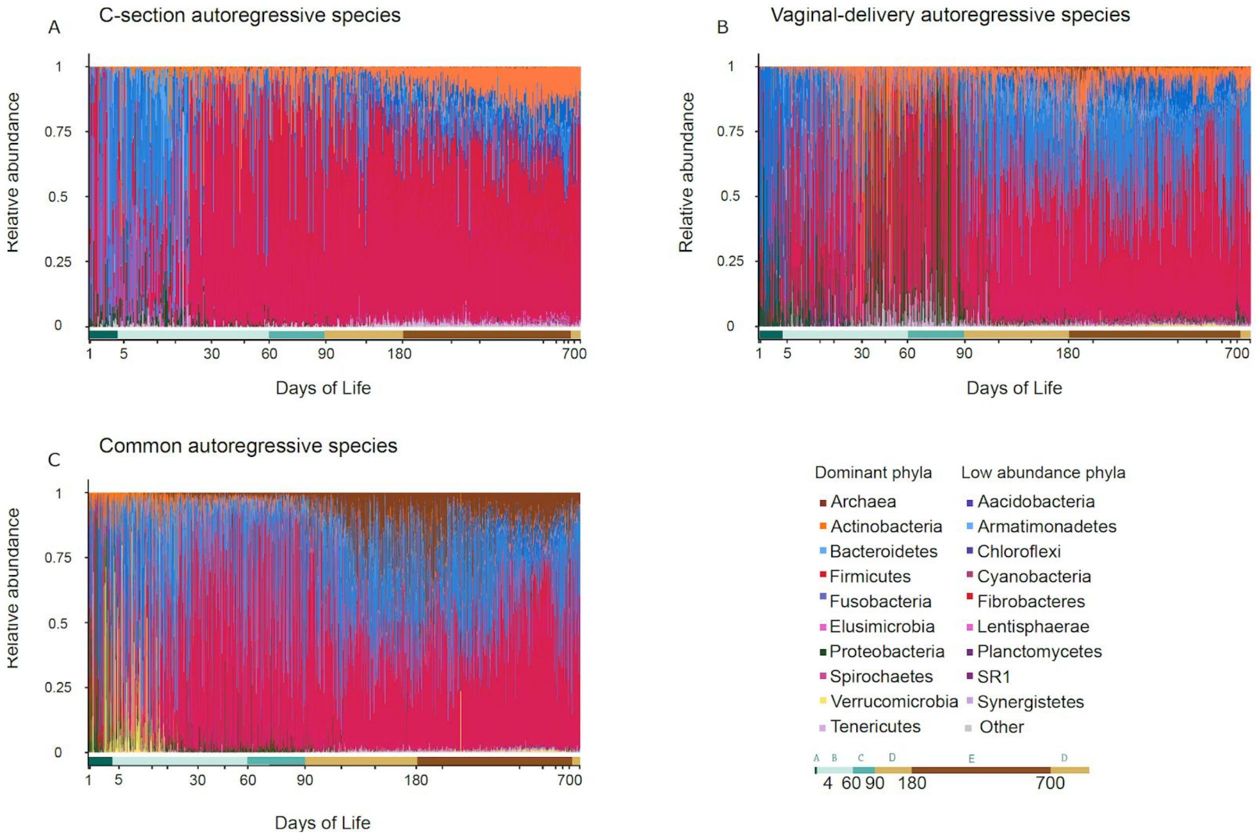

Supplementary Figure 18 - **Microbial dynamics of autoregressive species is delivery-mode dependent. A.** Relative abundance of the 50 microbial families for the 494 species found to be autoregressive only in C-section animals. Relative abundance values were re-calculated using these species only. Y-axis represents the re-calculated relative abundance, X-axis represents all C-section samples (n = 572), sorted by sampling day. All families belonging to the same phylum are colored by different shades of the same color. The main phyla are described at the bottom-right of the figure. **B.** Relative abundance of the 58 microbial families for the 494 species found to be autoregressive only in vaginally delivered animals. Relative abundance values were re-calculated using these species only. Y-axis represents the re-calculated relative abundance, X-axis represents all vaginally delivered samples (n = 1062), sorted by sampling day. **C.** Relative abundance of the 52 microbial families for the 401 species found to be autoregressive in both C-section and vaginally delivered animals. Relative abundance values were re-calculated using these species only. Y-axis represents the re-calculated relative abundance, X-axis represents all samples (n = 1634), sorted by sampling day. See Supplementary Figure 22 for the full family level color code. Source data is provided as a Source Data file.

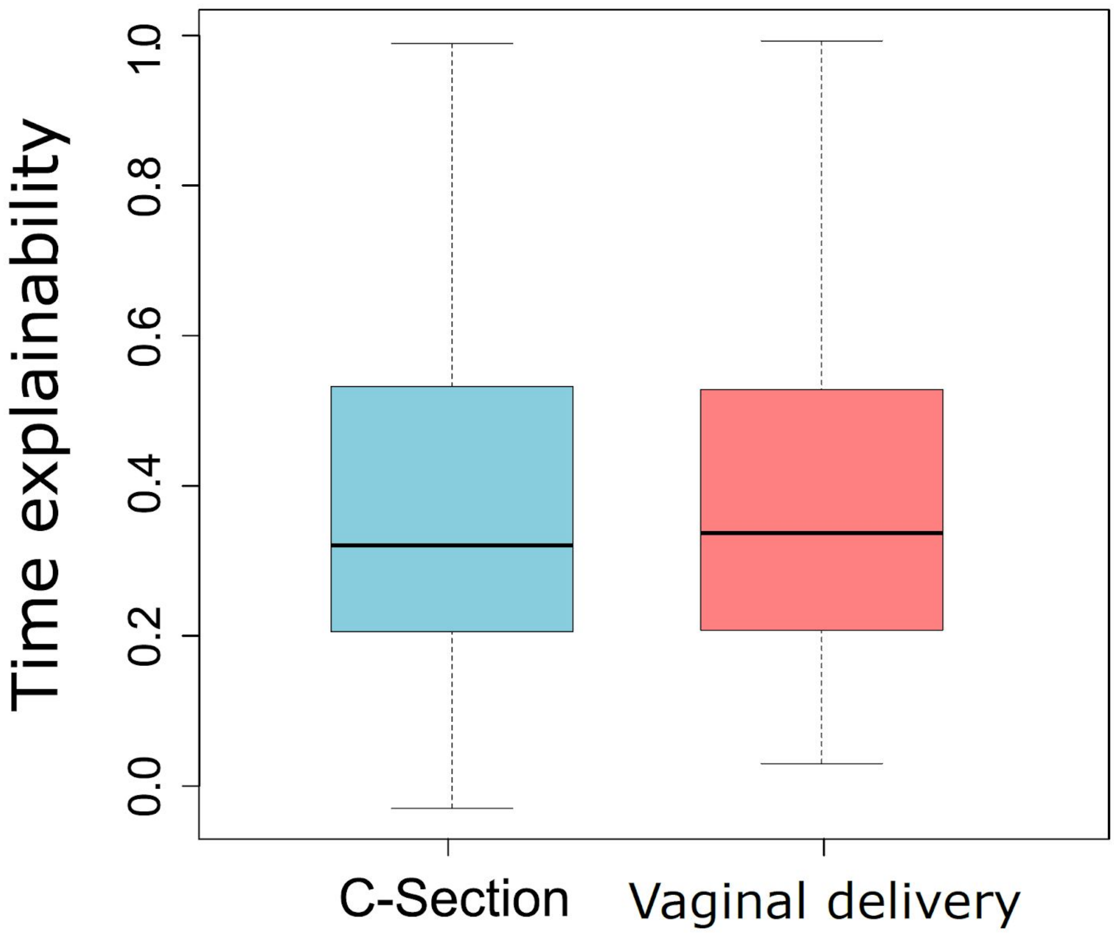

Supplementary Figure 19 - **Time explainability is similar for the two delivery-mode groups.** Time explainability is defined as the dependence of OTU (n = 494 OTUs for C-Section, n = 494 OTUs for Vaginal delivery) relative abundance on the previous time point's community composition (t-1) (see Methods). In the data shown as box plots, the horizontal line in the box represents the median and whiskers indicate the lowest and highest point within 1.5 interquartile ranges of the lower or upper quartile, respectively. Source data is provided as a Source Data file.

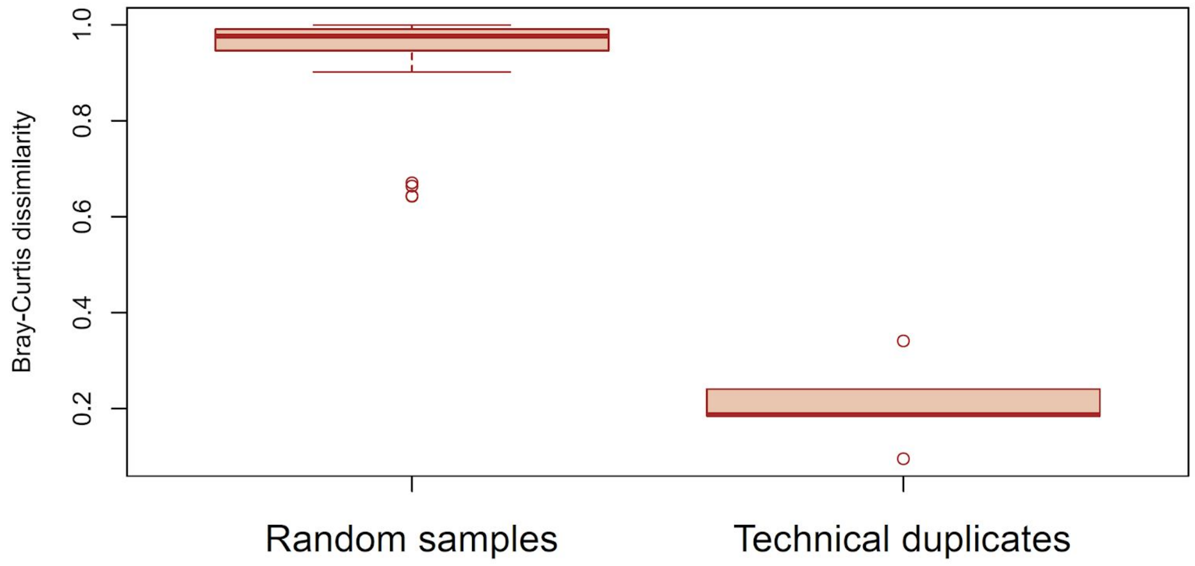

Supplementary Figure 20 - **Technical duplicates were more similar to each other than to other biological samples.** Bray-Curtis dissimilarity showed that technical duplicates (n = 5) were more similar to each other than to other random biological samples (t-test, P = $2.7*10^{-19}<0.001$, two sided test). Boxes represent the interquartile range (IQR) between the first and third quartiles (25th and 75th percentiles, respectively) and the horizontal line inside the box defines the median. Whiskers represent the lowest and highest values within 1.5 times the IQR from the first and third quartiles, respectively. Source data is provided as a Source Data file.

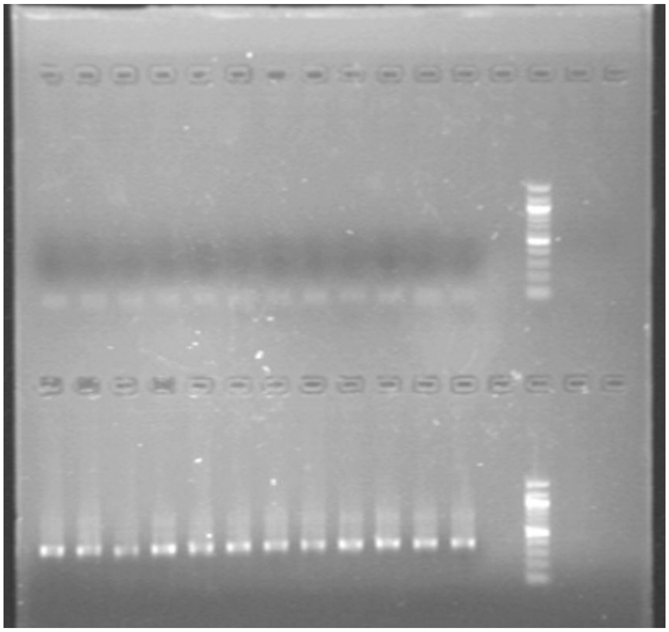

Supplementary Figure 21 - **Example for contamination check performed during product amplification.** DNA gel performed on PCR products from the extracted DNA of rumen samples. Top row in the gel represents control wells, and the bottom row represents PCR products of rumen samples.

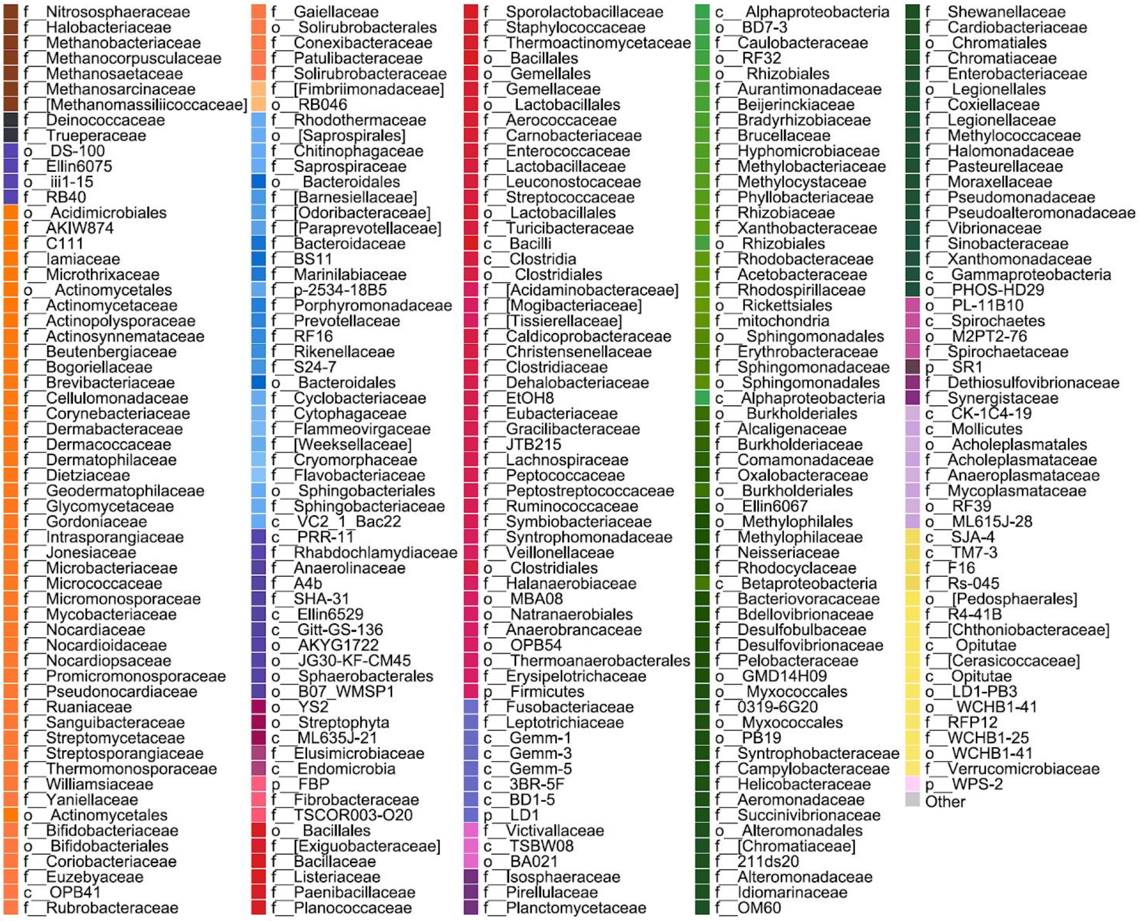

Supplementary Figure 22 - **Color code for all 291 microbial families.**

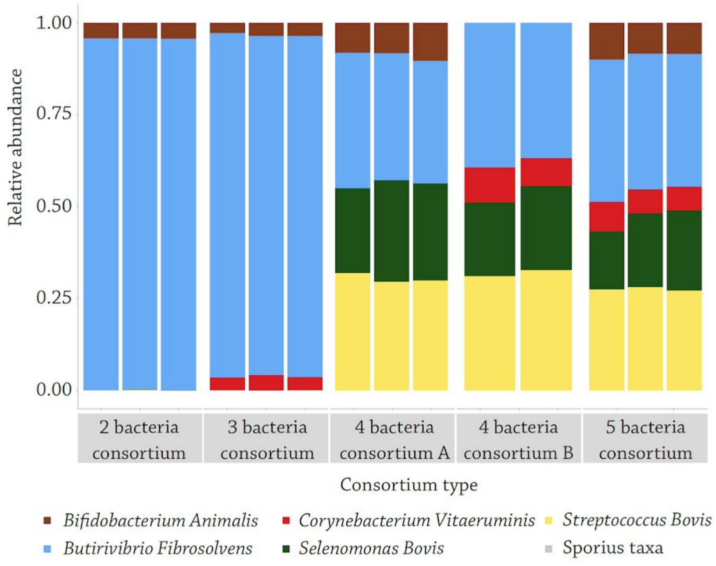

Consortium	Bacteria inserted	Bacteria detected
2-bacteria consortium	*Bifidobacterium animalis*	*Bifidobacterium animalis*
	Butyrivibrio fibrosolvens	*Butyrivibrio fibrosolvens*
3-bacteria consortium	*Bifidobacterium animalis*	*Bifidobacterium animalis*
	Butyrivibrio fibrosolvens	*Butyrivibrio fibrosolvens*
	Corynebacterium vitaeruminis	*Corynebacterium vitaeruminis*
4- bacteria A consortium	*Bifidobacterium animalis*	*Bifidobacterium animalis*
	Butyrivibrio fibrosolvens	*Butyrivibrio fibrosolvens*
	Selenomonas bovis	*Selenomonas bovis*
	Streptococcus bovis	*Streptococcus bovis*
4-bacteria B consortium	*Butyrivibrio fibrosolvens*	*Butyrivibrio fibrosolvens*
	Corynebacterium vitaeruminis	*Corynebacterium vitaeruminis*
	Selenomonas bovis	*Selenomonas bovis*
	Streptococcus bovis	*Streptococcus bovis*
5-bacteria consortium	*Bifidobacterium animalis*	*Bifidobacterium animalis*
	Butyrivibrio fibrosolvens	*Butyrivibrio fibrosolvens*
	Corynebacterium vitaeruminis	*Corynebacterium vitaeruminis*
	Selenomonas bovis	*Selenomonas bovis*
	Streptococcus bovis	*Streptococcus bovis*

Supplementary Figure 23 - **Relative abundance of sequenced bacteria within the different consortia**. Five different bacterial consortia were created, each consortium was repeated 3 times (except for the 4-bacteria consortium B). The adjacent table describes the bacteria inserted and identified in each consortium. Source data is provided as a Source Data file.
