# Supplementary material for: Stochasticity constrained by deterministic effects of diet and age drive rumen microbiome assembly dynamics
Source: Nat Commun. 2020 Apr 20;11:1904. doi: 10.1038/s41467-020-15652-8 (PMC7170844; doi:10.1038/s41467-020-15652-8)
Supplement: Supplementary file 2 — Description of Additional Supplementary Files [file 41467_2020_15652_MOESM2_ESM.pdf]

Description of Additional Supplementary Files

File Name: Supplementary Data 1
Description: Random Forest results for diets B and D based on supervised_learning.py on QIIME.

File Name: Supplementary Data 2
Description: Core OTU table.

File Name: Supplementary Data 3
Description: List of delivery mode associated species.

File Name: Supplementary Data 4
Description: Autoregressive species for each delivery mode found using MTV-LMM.

File Name: Supplementary Data 5
Description: - List of participating animals.

File Name: Supplementary Data 6
Description: Metadata table for all samples.

File Name: Supplementary Data 7
Description: Diet composition for diets B, C, D and E.

File Name: Supplementary Data 8
Description: Primers list

File Name: Supplementary Data 9
Description: Full OTU table in biom format.

File Name: Supplementary Data 10
Description: Contingency table for Chi square test for species appearing at day 1+2 only in one delivery mode.

File Name: Supplementary Data 11
Description: List of cows selected for each group in habitat associated species analysis.

File Name: Supplementary Data 12
Description: Probability table of each minor OTU to represent a sequencing error.

File Name: Supplementary Data 13
Description: OTUs that were found to be sequencing errors.
