# Supplementary material for: Stochasticity constrained by deterministic effects of diet and age drive rumen microbiome assembly dynamics
Source: Nat Commun. 2020 Apr 20;11:1904. doi: 10.1038/s41467-020-15652-8 (PMC7170844; doi:10.1038/s41467-020-15652-8)
Supplement: Supplementary file 16 — Reporting Summary [file 41467_2020_15652_MOESM16_ESM.pdf]

nature research

Corresponding author(s): Itzik Mizrahi

Last updated by author(s): Jan 31, 2020

Reporting Summary

Nature Research wishes to improve the reproducibility of the work that we publish. This form provides structure for consistency and transparency in reporting. For further information on Nature Research policies, see Authors & Referees and the Editorial Policy Checklist .

Statistics

For all statistical analyses, confirm that the following items are present in the figure legend, table legend, main text, or Methods section.

n/a	Confirmed	
☐	☒	The exact sample size (*n*) for each experimental group/condition, given as a discrete number and unit of measurement
☐	☒	A statement on whether measurements were taken from distinct samples or whether the same sample was measured repeatedly
☒	☐	The statistical test(s) used AND whether they are one- or two-sided<br>*Only common tests should be described solely by name; describe more complex techniques in the Methods section.*
☒	☐	A description of all covariates tested
☒	☐	A description of any assumptions or corrections, such as tests of normality and adjustment for multiple comparisons
☐	☒	A full description of the statistical parameters including central tendency (e.g. means) or other basic estimates (e.g. regression coefficient) AND variation (e.g. standard deviation) or associated estimates of uncertainty (e.g. confidence intervals)
☐	☒	For null hypothesis testing, the test statistic (e.g. *F*, *t*, *r*) with confidence intervals, effect sizes, degrees of freedom and *P* value noted<br>*Give P values as exact values whenever suitable.*
☒	☐	For Bayesian analysis, information on the choice of priors and Markov chain Monte Carlo settings
☐	☒	For hierarchical and complex designs, identification of the appropriate level for tests and full reporting of outcomes
☐	☒	Estimates of effect sizes (e.g. Cohen's *d*, Pearson's *r*), indicating how they were calculated

Our web collection on statistics for biologists contains articles on many of the points above.

Software and code

Policy information about availability of computer code

Data collection	No software was used to collect the data
Data analysis	Data analyses was performed using qiime 1.9 and R version 3.5<br>MTV - LMM was performed using the tool published by Shenhav et el., 2019.<br>All figures were constructed using the ggplot package in R .<br>All statistical analyses was performed using R.<br>Taxonomy assignment of species was performed using BLAST against the 16S rRNA reference database RDP (version 10)<br>Species were defined at 97% identity using UCLUST algorithm (Edgar et al., 2010, version 1.22q)<br>EMMAX version 20120210<br>PLINK version 1.07

For manuscripts utilizing custom algorithms or software that are central to the research but not yet described in published literature, software must be made available to editors/reviewers. We strongly encourage code deposition in a community repository (e.g. GitHub). See the Nature Research guidelines for submitting code & software for further information.

Data

Policy information about availability of data

All manuscripts must include a data availability statement. This statement should provide the following information, where applicable:

- Accession codes, unique identifiers, or web links for publicly available datasets
- A list of figures that have associated raw data
- A description of any restrictions on data availability

All sequencing files and metadata are deposited in SRA under PRJNA591750.

Field-specific reporting

Please select the one below that is the best fit for your research. If you are not sure, read the appropriate sections before making your selection.

☐ Life sciences ☐ Behavioural & social sciences ☒ Ecological, evolutionary & environmental sciences

For a reference copy of the document with all sections, see nature.com/documents/nr-reporting-summary-flat.pdf

Ecological, evolutionary & environmental sciences study design

All studies must disclose on these points even when the disclosure is negative.

Study description	We studied forces that drive the assembly of rumen microbiomes throughout a cow's life, with emphasis on the balance between stochastic and deterministic processes. We documented the development of the rumen microbiome from birth to adulthood in 45 animals (15 over a 3-year period) using 16S-rRNA amplicon gene data. We found that the animals shared a group of core successional species that invaded early on and persisted until adulthood. Along with deterministic factors, such as age and diet, early arriving species exerted strong priority effects, whereby dynamics of late successional taxa were strongly dependent on microbiome composition at early life stages. Priority effects also manifested as dramatic changes in microbiome development dynamics between animals delivered by C-section vs. natural birth, with the former undergoing much more rapid species invasion and accelerated microbiome development. Overall, our findings show that chance events early in life have a strong impact on development of animal microbiomes.
Research sample	We sampled the rumen content of 45 Holstein cows, bacterial DNA was extracted and sequenced for 16S rRNA gene sequencing. The rationale for choosing these animals was to examine microbial assembly in ruminants. We sampled both female and male animals, between the age of 0 days till up to 831 days
Sampling strategy	Sampling strategy was in accordance with Jami et al., 2013, sample size was calculated by exceeding the minimum number of animals required for rarefaction.
Data collection	Samples were collected using custom made stomach tube by O.F.
Timing and spatial scale	Samples were taken between 2013-2015, due to the higher variance in microbial community assembly, samples were taken weekly at the first six months of life, after-which samples were taken once a month. The experiment was conducted at the Volcani Center research experimental dairy farm, Rishon Letzion, Israel.
Data exclusions	No data was excluded.
Reproducibility	We ensured the reproducibility of our findings by performing the same sampling regime for multiple animals, thus strengthening our results. Animals were born in two time frames, each frame was 4 months long.
Randomization	Animals were randomly allocated into delivery mode groups, only healthy lactating cows were chosen to participate in this experiment.
Blinding	All analyses was performed after the sampling period, thus blinding it to the experimental setup.

Did the study involve field work? ☐ Yes ☒ No

Reporting for specific materials, systems and methods

We require information from authors about some types of materials, experimental systems and methods used in many studies. Here, indicate whether each material, system or method listed is relevant to your study. If you are not sure if a list item applies to your research, read the appropriate section before selecting a response.

Materials & experimental systems

n/a	Involved in the study	
☒	☐	Antibodies
☒	☐	Eukaryotic cell lines
☒	☐	Palaeontology
☐	☒	Animals and other organisms
☒	☐	Human research participants
☒	☐	Clinical data

Methods

n/a	Involved in the study	
☒	☐	ChIP-seq
☒	☐	Flow cytometry
☒	☐	MRI-based neuroimaging

Animals and other organisms

Policy information about studies involving animals; ARRIVE guidelines recommended for reporting animal research

Laboratory animals	The study did not involve laboratory animals.
Wild animals	The study did not involve wild animals.
Field-collected samples	The study did not samples collected in the field.
Ethics oversight	The experiment was conducted at the experimental dairy farm facility of the Volcani Center, Agricultural Research Organization (ARO), Israel, and was approved by the local ethics committee of the Volcani Center (approval number 412/12IL and 566/15IL).

Note that full information on the approval of the study protocol must also be provided in the manuscript.
